# NeissLock provides an inducible protein anhydride for covalent targeting of endogenous proteins

**DOI:** 10.1038/s41467-021-20963-5

**Published:** 2021-01-29

**Authors:** Arne H. A. Scheu, Sheryl Y. T. Lim, Felix J. Metzner, Shabaz Mohammed, Mark Howarth

**Affiliations:** 1 Department of Biochemistry, University of Oxford, South Parks Road, Oxford, OX1 3QU UK; 2 Present Address: Gene Center and Department of Biochemistry, Ludwig-Maximilians-Universität München, Feodor-Lynen-Straße 25, 81377 Munich, Germany

**Keywords:** Chemical modification, Protein design, Structural biology

## Abstract

*The Neisseria meningitidis* protein *FrpC* contains a self-processing module (SPM) undergoing autoproteolysis via an aspartic anhydride. Herein, we establish NeissLock, using a binding protein genetically fused to SPM. Upon calcium triggering of SPM, the anhydride at the C-terminus of the binding protein allows nucleophilic attack by its target protein, ligating the complex. We establish a computational tool to search the Protein Data Bank, assessing proximity of amines to C-termini. We optimize NeissLock using the Ornithine Decarboxylase/Antizyme complex. Various sites on the target (α-amine or ε-amines) react with the anhydride, but reaction is blocked if the partner does not dock. Ligation is efficient at pH 7.0, with half-time less than 2 min. We arm Transforming Growth Factor-α with SPM, enabling specific covalent coupling to Epidermal Growth Factor Receptor at the cell-surface. NeissLock harnesses distinctive protein chemistry for high-yield covalent targeting of endogenous proteins, advancing the possibilities for molecular engineering.

## Abstract (web summary)

Covalent conjugation of endogenous protein complexes offers many opportunities for fundamental and clinical research. Based on a bacterial protein domain that forms a reactive anhydride in the presence of Ca^2+^, the authors here develop a system that enables the covalent capture of endogenous binding partners.

## Introduction

Covalent conjugation to proteins presents unique opportunities. Compared to typical non-covalent coupling approaches, decoration through a stable covalent bond can enhance long-term imaging, biomaterial strength, therapeutic or vaccine efficacy, and diagnostic sensitivity^1–5^. Much attention has focused on peptide tags able to react with protein partners^6^, e.g., SpyTag, split inteins, sortase or OaAEP1, or reactions through click chemistry pairs^7^. However, distinct strategies are required to react with unmodified endogenous proteins, which has greater relevance for therapeutic settings. For this challenge, proximity-directed ligation has been an important approach, employing either small molecules or protein binders^4,8^. Small molecules with affinity for a target protein may be equipped with reactive functionalities, favoring covalent reaction with nearby nucleophiles in the binding site (particularly Cys but also extended to other protein side-chains)^4^. This approach has been successful for certain proteins, particularly those with deep and unique pockets facilitating specific ligand binding^4^. To generalize this approach to a wider range of protein targets, one would ideally harness natural or engineered proteins and confer on them covalent reactivity with their specific protein partners. We previously applied this strategy through chemical attachment of the weak electrophile acrylamide, for covalent ligation by an affibody to an apposed nucleophile on its cognate partner^9^. Since then, various electrophilic unnatural amino acids have been genetically encoded for coupling to endogenous proteins^10–14^. However, it would be preferable to obtain such reactivity using standard amino acids and with inducible reaction.

**Fig. 1:**
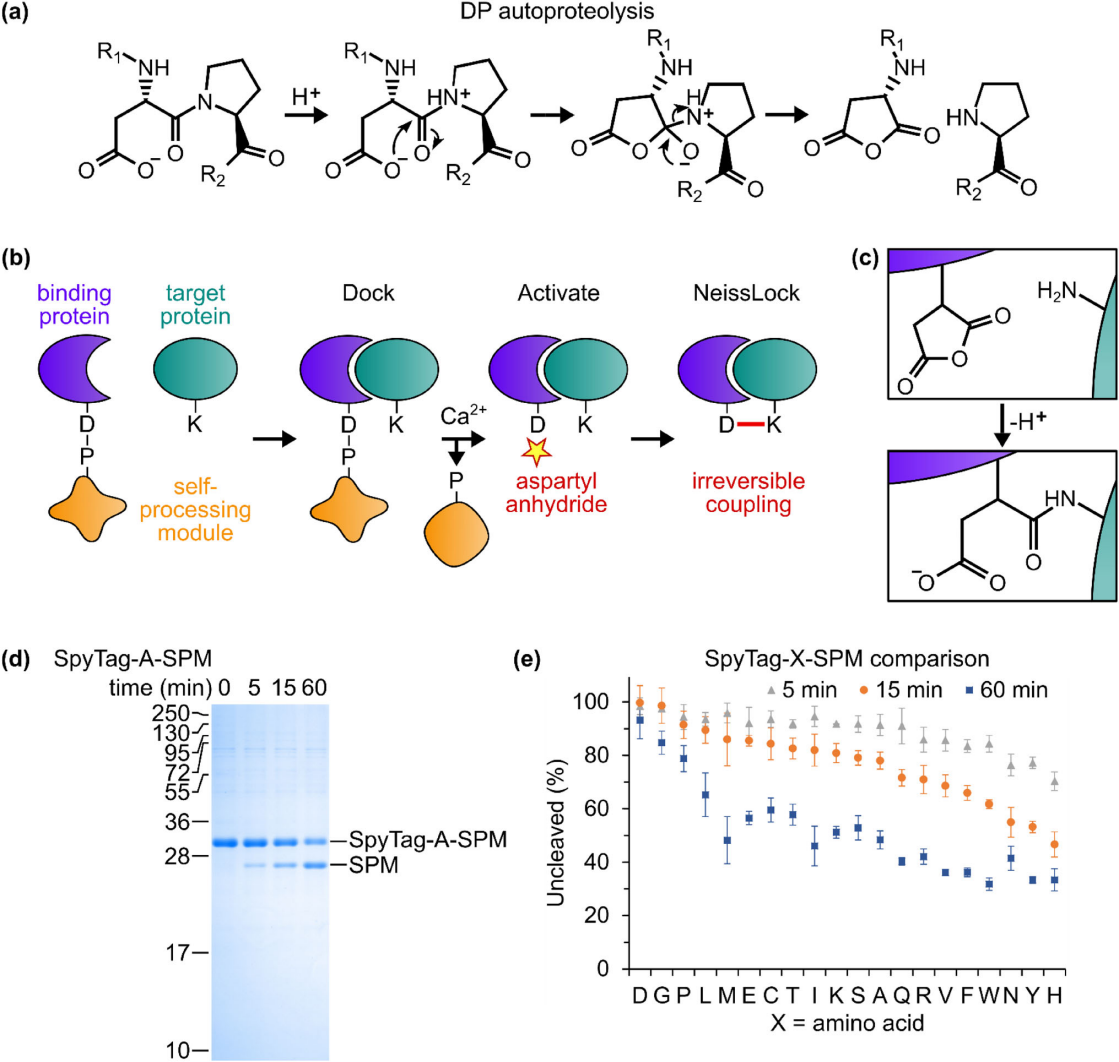
The NeissLock concept and optimizing autoproteolysis. **a** The FrpC self-processing module (SPM) catalyzes autoproteolytic cleavage at an Asp-Pro peptide bond, induced by calcium, generating an anhydride. **b** NeissLock vision. SPM (orange) is recombinantly fused to a binding protein (blue) which docks with the target protein (green). Adding calcium promotes generation of the anhydride (yellow star), so the binding protein can form a covalent bond to the target protein. **c** Schematic of reaction of amine on target protein with aspartic anhydride on binding protein. **d** Time-course of SPM cleavage with Ala preceding Asp-Pro, analyzed by SDS-PAGE with Coomassie staining (pH 7.4, 37 °C). Molecular weight markers represent kDa. **e** Histogram of SPM cleavage rate with each residue before Asp-Pro, moving from the least cleaved residue at 15 min on the left to the most cleaved residue on the right (mean of triplicate ± 1 s.d.; some error bars are too small to be visible; pH 7.4 and 37 °C. Source data are provided as a Source Data file.

FrpC from *Neisseria meningitidis* is a secretory protein containing a self-processing module (SPM), which displays calcium-dependent autoproteolytic activity at an aspartate-proline dipeptide^15,16^. Moving from the low calcium environment inside the cell (Ca^2+^ ~ 0.1 µM) to the extracellular medium (Ca^2+^ 1–2 mM)^17^, calcium-dependent conformational change in SPM mediates FrpC processing^18–20^. Autoproteolysis follows protonation of the main-chain nitrogen of proline, leading to formation of an aspartic anhydride at the newly generated C-terminus^16,21^ (Fig. 1a). Importantly, the FrpC region N-terminal to the aspartate-proline cleavage site (FrpC1-414) is not required for autoproteolysis, so that SPM retains activity when recombinantly fused to the C-terminus of various proteins^16,21,22^. With a cleavage scar of a single amino acid and mild conditions for activation, previous SPM-derived technologies focused on fusion-protein purification^21,22^. For protein purification, the presence of the anhydride intermediate is an unwelcome side-effect, needing to be quenched by excess free nucleophile^21,22^. On the contrary, we saw this anhydride as a unique opportunity.

In this work, we harness the anhydride intermediate formed by SPM to establish inducible intermolecular ligation. In our approach, a binder protein bearing SPM docks with its target protein, Ca^2+^ induces anhydride formation, and a nearby nucleophile on the target protein reacts with the anhydride to lock the complex irreversibly (Fig. 1b, c). We call this approach NeissLock (Fig. 1b) and herein present demonstration of this technology for specific and rapid protein targeting in vitro and on living cells.

## Results

### SPM efficiency depended on the residue preceding the cleavage site

It was previously reported that the fusion protein had a major effect on the cleavage efficiency of SPM, but it was unclear which features were important^21,22^. We hypothesized that the residue preceding the cleavage site was key. Therefore, we fused SPM to the unstructured SpyTag peptide^23^ and tested the impact on the cleavage rate of each of the 20 amino acids in front of the reactive aspartate-proline dipeptide (SpyTag-X-SPM) (Fig. 1d, e). Protein constructs were all expressed solubly in *Escherichia coli* and purified using Ni-NTA. Each purified protein was then incubated with high Ca^2+^ at 37 °C for 5, 15, or 60 min. Reaction was stopped by addition of EDTA and boiling in sodium dodecyl sulfate (SDS), ahead of SDS-PAGE with Coomassie staining (Fig. 1d, e). When X was D, G, or P, there was minimal cleavage even after 60 min (Fig. 1e). H, Y, and W were most efficient, with Y being the residue here in native FrpC (Fig. 1e). Therefore, for the development of NeissLock, we chose to use Y preceding the aspartate-proline at the fusion site.

### Computational analysis of the Protein Data Bank for NeissLock

**Fig. 2:**
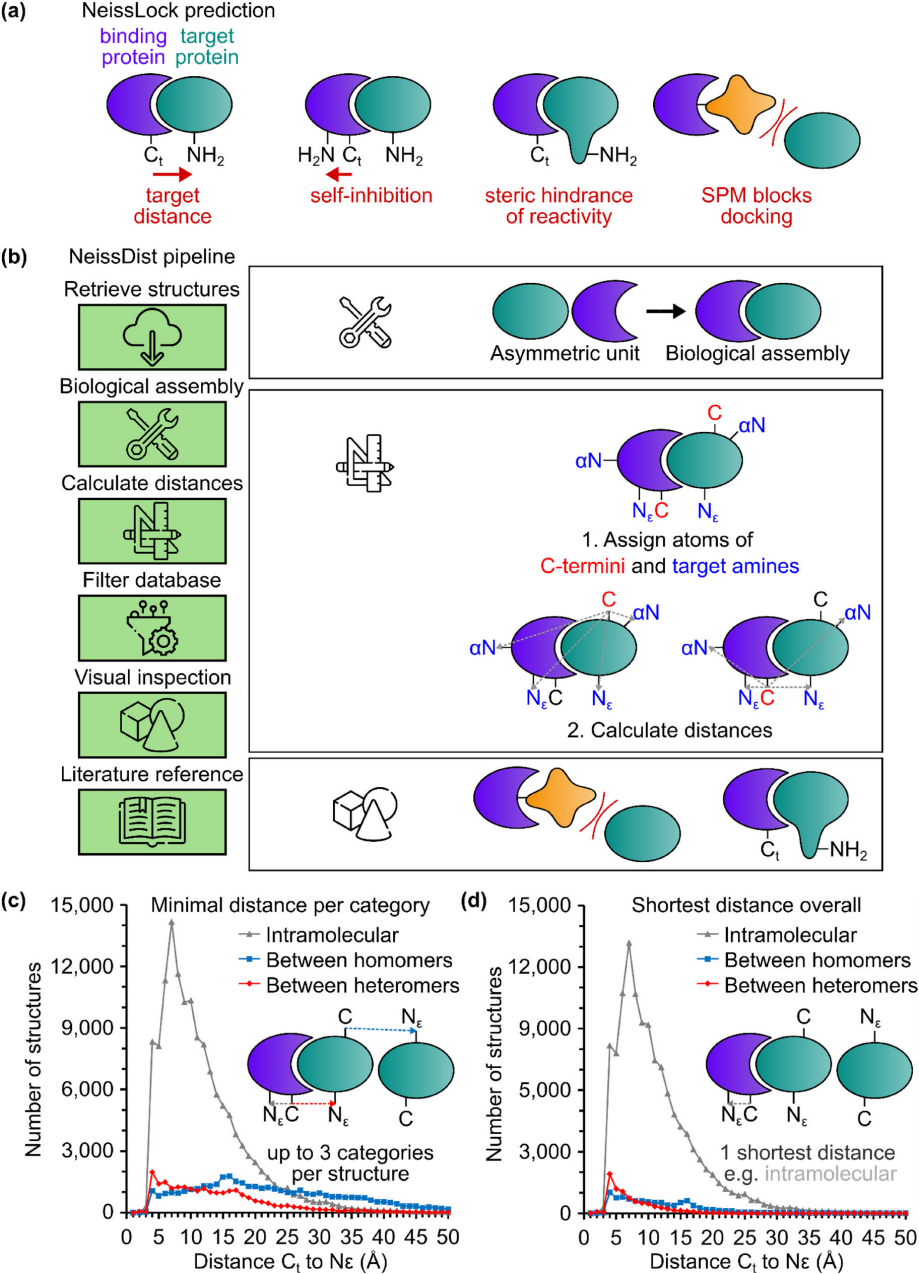
Software for selection of NeissLock complexes. **a** Considerations for NeissLock complex selection. (i) The target protein (green) should have an α- or ε-amine proximal to the C-terminus (C_t_) of the binder protein (blue). (ii) To avoid self-reaction, the binder protein should not feature an amine close to its own C-terminus. (iii) A bulky region on the target protein may sterically impede amine reactivity. (iv) SPM should not occlude the interface between binder and target proteins. **b** NeissDist database pipeline. **c** Minimal distances per category from NeissDist. Only C-terminus (C_t_) to lysine with a primary distance from C (main-chain carbonyl carbon) to Nε was considered (see methods for N-terminal lysines). Count of those distances below distance cut-off (Å) for all types. **d** Comparison of those primary distances from NeissDist to identify shortest distance per structure. Count of these distances below distance cut-off (Å) according to type.

**Fig. 3:**
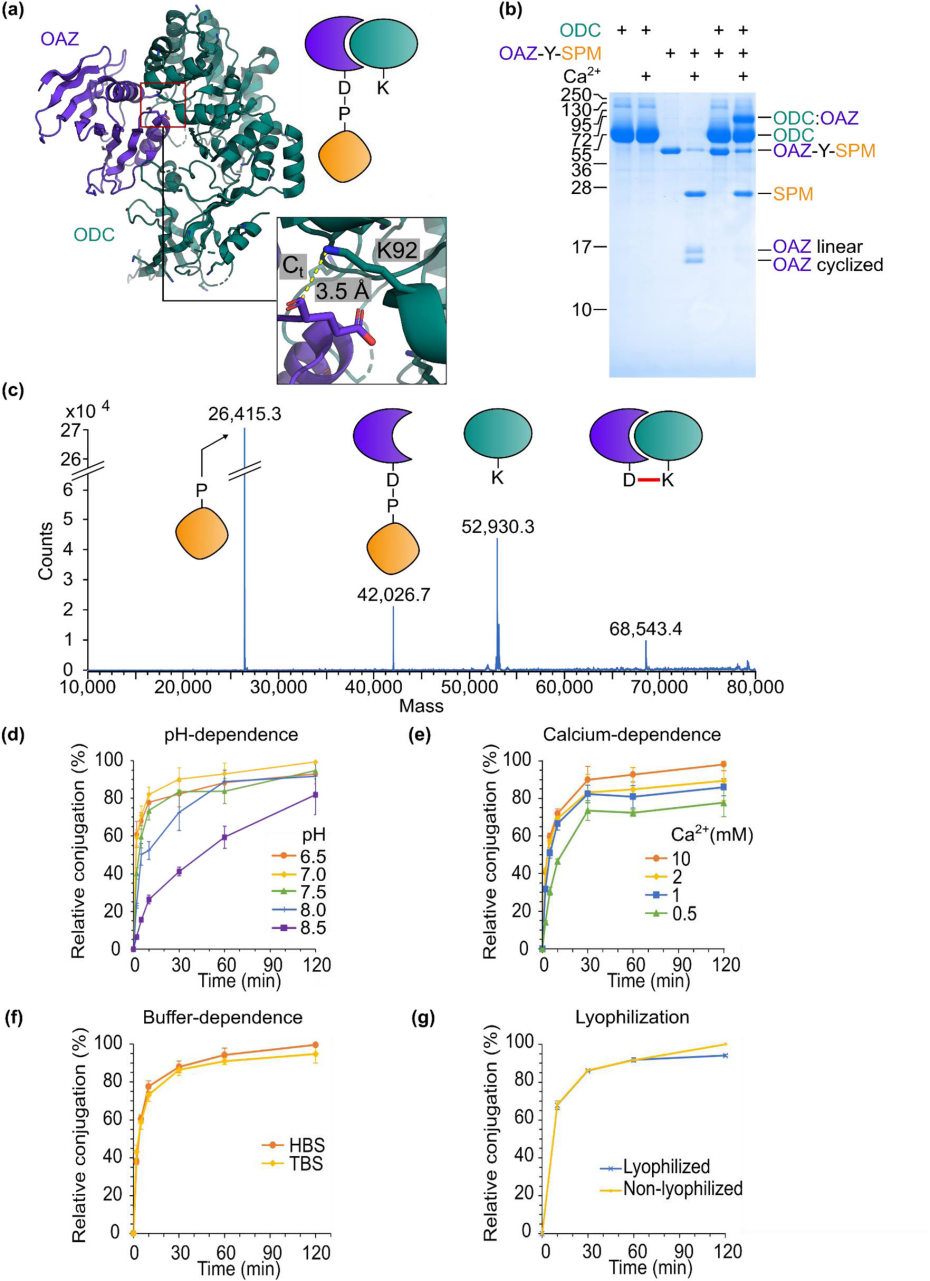
Establishment of covalent bond formation by NeissLock. **a** Crystal structure of ODC/OAZ complex (PDB 4zgy). Inset shows the proximity from K92 of ODC to the C-terminal carbonyl carbon of OAZ, close to where the anhydride should be. **b** ODC reacted covalently with OAZ. 30 µM ODC and 10 µM OAZ-Y-SPM were incubated for 16 h with or without Ca^2+^, boiled in SDS-loading buffer, and analyzed by SDS-PAGE with Coomassie staining. Molecular weight markers represent kDa. **c** Intact protein electrospray ionization MS confirms covalent coupling of OAZ to ODC. Analysis of products from incubation of ODC with OAZ-Y-SPM in the presence of Ca^2+^. **d** pH-dependence of conjugation. OAZ-GSY-SPM was incubated with ODC and Ca^2+^ at the indicated pH (compared to pH 7.0 at 120 min). **e** Calcium-dependence of conjugation. OAZ-GSY-SPM was incubated with ODC and Ca^2+^ at the indicated concentrations (conjugation relative to 10 mM Ca^2^). **f** Buffer-dependence of conjugation. OAZ-GSY-SPM was incubated with ODC at pH 7.4 and 10 mM Ca^2+^ in HBS or TBS (conjugation relative to HBS). **g** Reactivity was unaffected by lyophilization. ODC was incubated with lyophilized or non-lyophilized OAZ-GSY-SPM. (All proteins 10 µM at 37 °C and mean of triplicate ± 1 s.d.). Source data are provided as a Source Data file.

For successful conjugation via NeissLock, we predicted the following requirements: (1) a short distance between the C-terminal anhydride of the binding protein and the nearest accessible nucleophile on the target protein, (2) SPM should not sterically disrupt docking between the binding protein and target protein, and (3) avoidance of an own-goal (self-reaction where a nucleophile on the binding protein rather than on the target protein reacts with the anhydride) (Fig. 2a). Therefore, we developed a computational approach to search the Protein Data Bank (PDB) to identify protein-protein complexes with suitable residue distances. This program was called NeissDist (Fig. 2b). For each protein structure in the PDB, we aimed to generate a set of distances from the most distal resolved residue in each polypeptide to each reactive amine. We classified distances according to whether they were intramolecular, between homomers, or between heteromers (Fig. 2c). We also classified which of these distances was shortest in each structure (Fig. 2d). The protein database was not previously reduced by biological parameters (e.g., sequence homology), to increase the chance to identify candidates. For instance, with these parameters, 9793 structures were annotated to feature an intermolecular distance between heteromers from C_t_ to lysine ε-amine <10 Å (Fig. 2c) (out of 140,016 structures for which a distance was determined). This distance was the shortest overall distance from C_t_ to lysine ε-amine in 6,565 structures (Fig. 2d). We shortlisted structures after visualization and inspection in PyMOL. A sample of hits is illustrated in Supplementary Table 1 and the set of 6,497 hits is described in Supplementary Data 1. Combining promising structural characteristics with simple expression from *E. coli*, we selected as our model system the complex between Ornithine Decarboxylase (ODC) and Ornithine Decarboxylase Antizyme (OAZ) (PDB 4zgy)^24^ (Supplementary Table 1, Fig. 3a). ODC catalyzes the rate-limiting step in the biosynthesis of polyamines, which has key effects on the growth and differentiation of cells. OAZ binding blocks the activity and promotes the degradation of ODC in cells^24^.

### Establishing the NeissLock principle

To establish a covalent ODC/OAZ complex, we initially optimized the SPM sequence. The boundaries of SPM in the literature (including the reactive aspartate-proline) vary from amino acid residues 414–657^16,21,22^ to 414–591^18–20^ (Supplementary Fig. 1a). We predicted SPM to range until residue 584 using the Ginzu domain prediction method implemented on the Robetta server^25^. However, we performed further step-wise truncation of SPM according to the secondary structure predicted with the JPred4 server^26^ (Supplementary Fig. 1a). We found that the shortened form of SPM (FrpC414–591) showed a reduced rate of calcium-induced cleavage compared to forms ending at 613, 635, or 657 (Supplementary Fig. 1b). Balancing expression yield and purity with reaction speed, we settled on FrpC414–657 for our SPM.

In the ODC/OAZ crystal structure, the last resolved residue of OAZ, E219 (carbonyl carbon), is 3.5 Å from ODC K92′s ε-amine (atom Nε)^24^. We predicted this short distance to be favorable for conjugation (Fig. 3a). Following our NeissLock design criteria (Fig. 2a), E219 appeared to be sterically accessible to K92 in the complex, as well as far from a lysine on OAZ itself (nearest: K153 Nε 16.8 Å away). OAZ was truncated according to the modeled structure (95–219)^24^ and expressed with a Y spacer to enhance SPM reactivity. Therefore, OAZ-Y-SPM was our initial ODC-targeting NeissLock-probe.

Upon addition of calcium, OAZ-Y-SPM underwent self-processing to yield SPM and two OAZ species of differing mobility (Fig. 3b). These forms correspond to a linear OAZ species from hydrolysis and a cyclized species from self-reaction, i.e., intramolecular reaction of the anhydride with a nucleophile on OAZ, as determined by mass spectrometry (MS) (Supplementary Fig. 2). When ODC was mixed with OAZ-Y-SPM in the presence of Ca^2+^, an efficient reaction occurred between OAZ and ODC, with little free OAZ remaining (Fig. 3b). This covalent conjugation was validated by electrospray ionization MS. After OAZ-Y-SPM self-processing, we identified masses corresponding to SPM (calculated 26,415.1; observed 26,415.3), OAZ-Y-SPM (calculated 42,024.7; observed 42,026.7), ODC (calculated 52,929.9; observed 52,930.3) and ODC:OAZ conjugate (calculated 68,539.4; observed 68,543.4) (Fig. 3c).

### A flexible linker to SPM enhanced the cleavage and conjugation

With ODC/OAZ as a promising model system, we explored the parameters determining SPM cleavage and NeissLock conjugation. First, we noted that OAZ-Y-SPM displayed reduced cleavage compared to SpyTag-Y-SPM (Fig. 1e), while conjugation of OAZ-Y-SPM to ODC was similarly slow (Supplementary Fig. 1c, d). Steric hindrance was previously proposed as a reason for reduced cleavage rate in SPM-fusions^21^. We introduced a GS-linker into OAZ-Y-SPM to test its effect on cleavage rate and conjugation efficiency. With OAZ-GSY-SPM we obtained a substantial increase in cleavage rate and now rapid reaction with ODC (Supplementary Fig. 1d).

### Condition-dependence of NeissLock

We explored the optimal conditions for NeissLock reaction. ε-amines have an average pK_a_ of 10.5 in proteins^27^. Since only the deprotonated form of the ε-amine is nucleophilic and ε-amine would mostly be protonated at neutral pH, it was important to test at which pH the NeissLock approach was most effective. Testing between pH 6.5 and 8.5, cleavage was fastest at pH 6.5 or 7.0 and but still readily occurred up to pH 8.5 (Supplementary Fig. 1e). When testing NeissLock reaction, we were surprised to find that the rate of conjugation to ODC was highly efficient from pH 6.5 to 7.5 (Fig. 3d). At pH 7.0, the half-time of reaction was less than 2 min (Fig. 3d).

To confirm the role of the D414 residue, which was predicted to form the aspartic anhydride in SPM, D414A mutation in OAZ-GSY-SPM abolished both calcium-induced cleavage and covalent conjugation to ODC (Supplementary Fig. 3).

Previously, it was shown that the cleavage rate of SPM in fusion proteins was enhanced with higher calcium concentrations but still highly efficient at 2 mM Ca^2+^ ^20^. In order to probe the calcium-dependence of NeissLock, we tested between 0.5 and 10 mM Ca^2+^. Although conjugation to ODC was fastest at 10 mM Ca^2+^, we found that coupling was still efficient even at 0.5 mM (78% coupling yield compared to 10 mM Ca^2+^ after 2 h, Fig. 3e). The rapid coupling even at 0.5 mM Ca^2+^ suggests that physiological extracellular Ca^2+^ concentrations (1–2 mM)^17^ are sufficient for the NeissLock approach.

We then tested NeissLock’s compatibility with different buffers by incubating OAZ-GSY-SPM and ODC in HEPES-buffered saline (HBS) or Tris-buffered saline (TBS). The rate of conjugation to ODC was similar in HBS and TBS (95% coupling yield in TBS compared to HBS after 2 h, Fig. 3f). Phosphate-buffered saline (PBS) is inadvisable because the addition of calcium to activate SPM will lead to calcium phosphate precipitation.

To test if NeissLock is resilient to harsh treatments such as lyophilization, which may damage protein folding, we tested the conjugation rate of OAZ-GSY-SPM to ODC after reconstitution post-lyophilization. We found that even after lyophilization, SPM retained efficient coupling activity. There was little difference in coupling yield of the lyophilized sample (51 ± 3% yield, *n* = 3) compared to the untreated control (48 ± 3% yield, *n* = 3) after 2 h (Fig. 3g).

### NeissLock depended on initial non-covalent interaction

**Fig. 4:**
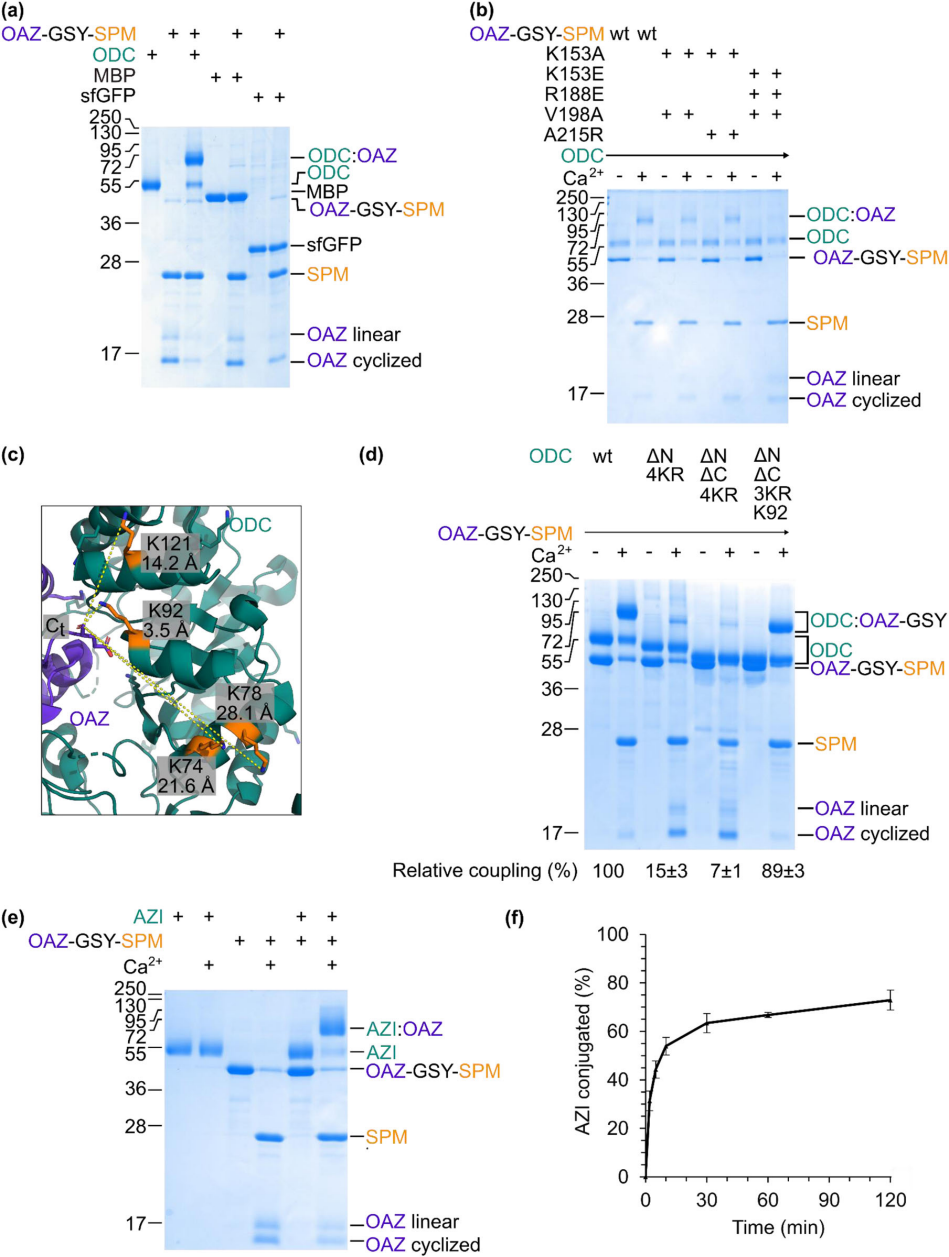
Investigation of OAZ NeissLock reaction. **a** Specific reaction by NeissLock. OAZ-GSY-SPM was incubated with Ca^2+^ for 16 h with ODC or non-cognate MBP or sfGFP (each protein at 10 µM). Samples were analyzed by SDS-PAGE with Coomassie staining. **b** Decreasing OAZ/ODC binding affinity blocked conjugation. Wild-type (wt) or point mutants of OAZ-GSY-SPM were incubated ± Ca^2+^ with ODC for 60 min with each protein at 0.5 µM, before SDS-PAGE with Coomassie staining. **c** Neighboring lysines on ODC. ODC/OAZ complex (PDB 4zgy) with C_t_ and nearby lysines (orange) shown in stick format. **d** Different sites on ODC reacted with OAZ. Potential crosslinking sites were removed from ODC to give ∆N (N-terminus truncated), ∆C (C-terminus truncated), and 4KR (K74R, K78R, K92R, K121R). Lysine was individually re-introduced at K92. OAZ-GSY-SPM was incubated with the indicated ODC mutant for 16 h at 37 °C ± Ca^2+^, before SDS-PAGE with Coomassie staining. Relative coupling compared to wt was quantified (mean of triplicate ± 1 s.d.). **e** Efficient NeissLock coupling to AZI. 2.5 µM AZI was incubated with 2.5 µM OAZ-GSY-SPM for 18 h at 37 °C ± Ca^2+^, before SDS-PAGE with Coomassie staining. **f** Rapid AZI coupling. 2.5 µM AZI was incubated with 2.5 µM OAZ-GSY-SPM in HEPES buffer, pH 7.4 at 37 °C with Ca^2+^. Coupling to AZI was quantified from SDS-PAGE with Coomassie (mean of triplicate ± 1 s.d.). Molecular weight markers represent kDa. Source data are provided as a Source Data file.

To determine whether OAZ-GSY-SPM would react non-specifically, we tested conjugation of OAZ-GSY-SPM to proteins expected to have no interaction with OAZ, i.e., maltose binding (MBP) or superfolder green fluorescent protein (sfGFP). At 10 µM each of OAZ-GSY-SPM and added protein, there was only trace conjugation to MBP (2.7 ± 0.3%, *n* = 3, compared to OAZ:ODC) and no observable conjugation to sfGFP (Fig. 4a).

Next, we tested whether a high affinity interaction was required for NeissLock. To facilitate affinity measurements by surface plasmon resonance (SPR), we introduced an AviTag to OAZ-GSY-SPM for site-specific biotinylation, allowing anchoring to the streptavidin-chip. We also introduced C175A in OAZ to reduce aggregation. We tested two mutations reported to reduce binding based on the mouse ODC/OAZ complex^28^, to generate the low affinity probe AviTag-OAZ K153A, V198A-GSY-SPM (mutations marked on the crystal structure in Supplementary Fig. 4a). Based on the crystal structure^24^, we also designed OAZ K153A, A215R-GSY-SPM to decrease ODC binding affinity. Upon conjugation with ODC, both mutants showed slightly reduced conjugation efficiency (Fig. 4b and Supplementary Fig. 4b; coupling yields were decreased by the presence of the AviTag.) Using SPR, we determined the K_d_ to ODC of wt AviTag-OAZ-GSY-SPM as 0.12 µM. The K_d_ for the K153A, V198A mutant was 25 µM and for OAZ K153A, A215R was 15 µM (Supplementary Fig. 4c–g). With these K_d_ values, one would still expect substantial docking of the OAZ mutants with ODC at the tested concentration (10 µM). Therefore, we incorporated charge inversion mutations at R188 and K153 of OAZ (Supplementary Fig. 4a), to decrease further the affinity for ODC. Binding of OAZ K153E, R188E, V198A-GSY-SPM to ODC was undetectable in SPR, indicative of a K_d_ > 100 µM (Supplementary Fig. 4g). Upon addition to ODC in the presence of Ca^2+^, there was minimal detectable crosslinking by this non-binding mutant, OAZ K153E, R188E, V198A-GSY-SPM (Fig. 4b). In summary, OAZ reacts with ODC by NeissLock in an affinity-dependent manner.

To test the specificity of NeissLock coupling in complex protein mixtures, A431 cell lysate was mixed with OAZ-GSY-SPM in the presence of calcium. The labeling pattern of OAZ was detected by an anti-His Western blot, detecting His_6_-tags at the N-termini of OAZ and ODC (Supplementary Fig. 5). We found that SPM retained efficient cleavage activity in cell lysate. We did not observe coupling by OAZ to other endogenous proteins present in the cell lysate (Supplementary Fig. 5). When an equimolar ratio of ODC was added to the cell lysate, a single band corresponding to the ODC:OAZ conjugate was observed (Supplementary Fig. 5). This suggests that NeissLock retained the ability to react with its cognate target even in complex environments.

### Various amines on the target can react with the binding protein

We identified OAZ as a model NeissLock probe based on the proximity of OAZ’s distal resolved residue E219 to ODC K92 (Fig. 3a). We, therefore, hypothesized that crosslinking primarily occurred at ODC K92. Using tryptic digest and liquid chromatography tandem mass spectrometry (LC-MS/MS) of OAZ-Y-SPM conjugated to ODC, we were able to identify a crosslinked peptide from reaction of K92 with the C-terminus of OAZ (Supplementary Fig. 6a). However, when we made ODC K92R, we were surprised to retain high amounts of covalent conjugate (Supplementary Fig. 6b). This conjugation suggests that K92 is a primary crosslink site, but that there are alternative crosslinking sites on ODC. Using ODC K92R, through tryptic digest and tandem MS, we identified another crosslink site from K121 to OAZ (Supplementary Fig. 6c, d).

To locate additional crosslinking sites on ODC, we used rational mutagenesis. First, we mutated all the ε-amines in proximity to OAZ’s C-terminus (Fig. 4c) to make ODC 4KR (ODC K74R K78R K92R K121R). However, ODC 4KR still coupled efficiently to OAZ (Supplementary Fig. 7a). Thereupon our suspicions were directed to the α-amine of ODC. The N-terminal region of ODC was unresolved in PDB 4zgy^24^. Although the first resolved N-terminal residue of ODC in the structure faces outwards, far from the ODC/OAZ interface, the crystal structure of the ODC homodimer (PDB 1d7k)^29^ suggested that the N-terminus of ODC might extend back towards the OAZ interface. Based on PDB 1d7k, we, therefore, removed the N-terminal His_6_-tag as well as 9 flexible residues from ODC 4KR, to generate ODC ΔN 4KR. Incubation of OAZ-GSY-SPM with ODC ΔN 4KR yielded only small amounts of conjugation (15% compared to wild-type ODC, Fig. 4d). ODC ΔN 4KR had no decrease in non-covalent binding to OAZ-GSY-SPM by SPR (Supplementary Fig. 4d, g).

Starting from the largely unreactive ODC ΔN 4KR, we were able to test the reaction efficiency of lysine at different sites on the protein surface. Re-introduction of K78 into ODC did not lead to substantial conjugation (Supplementary Fig. 7a). Re-introduction of K74 led to a low level of conjugation. Re-introduction of K92 or K121 rescued high levels of conjugation (Supplementary Fig. 7a), supporting the potency of these residues in reaction with the anhydride.

To locate the remaining crosslinking site(s) in ODC ΔN 4KR, we introduced a further truncation of the C-terminus of ODC that was unresolved in PDB 4zgy^24^, giving ODC ΔN ΔC 4KR (now ending at residue 421 of ODC). There are multiple nucleophilic residues within the unresolved C-terminus of ODC that are potential reactive sites. Incubation of OAZ-GSY-SPM with ODC ΔN ΔC 4KR now made conjugation barely detectable (Fig. 4d). Re-introduction of K92 rescued high levels of conjugation (89 ± 3% compared to wild-type ODC, *n* = 3, Fig. 4d). In summary, the OAZ anhydride conjugates efficiently with multiple different crosslinking sites on ODC.

To understand if OAZ self-interaction played a role in these crosslinking patterns, we tested the solution behavior of OAZ-GSY-SPM by size exclusion chromatography with multi-angle light scattering (SEC-MALS). This analysis gave a close correspondence between the predicted and observed M_w_ for a monomeric protein (Supplementary Fig. 7b).

To test the generality of NeissLock through OAZ, we evaluated conjugation of OAZ-GSY-SPM to human Antizyme Inhibitor (AZI). AZI binds with high affinity to OAZ, so reducing OAZ’s inhibition of ODC and leading to increased cellular polyamine levels which may favor tumor progression^24,30^. AZI has a well-positioned lysine for reaction with OAZ-GSY-SPM (Supplementary Fig. 8)^30^. When AZI was mixed with OAZ-GSY-SPM in the presence of Ca^2+^, we found highly efficient covalent coupling between OAZ and AZI (80 ± 3% yield, *n* = 3) (Fig. 4e). The covalent coupling was rapid and more than 50% of AZI was conjugated within 10 min (Fig. 4f).

### Anhydride reactivity with different nucleophiles

**Fig. 5:**
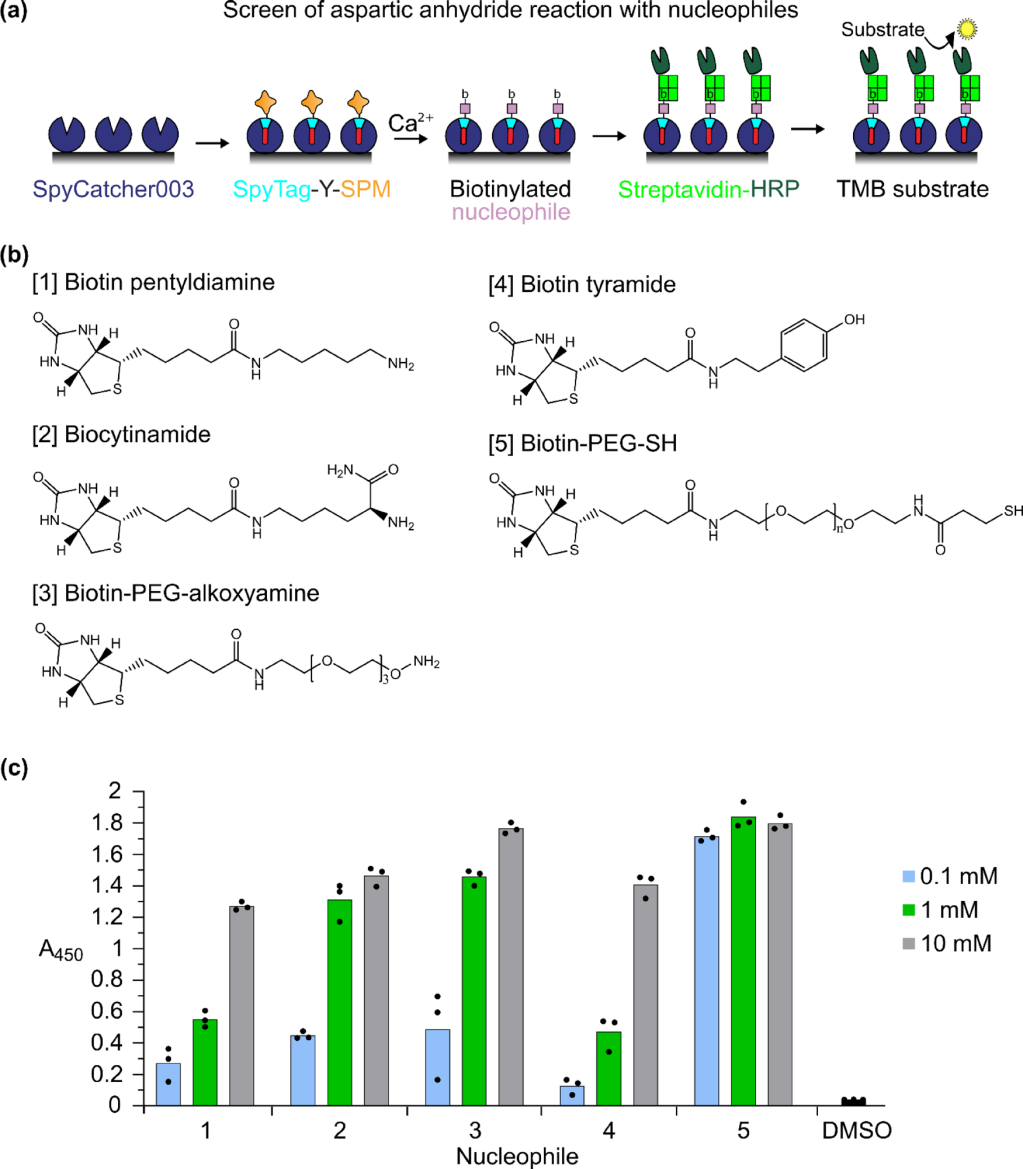
Investigation of anhydride reactivity. **a** Schematic of assay for assessing anhydride reactivity. A SpyCatcher003 (dark blue) fusion is immobilized on a microtiter plate. SpyTag (cyan) linked to SPM (orange) forms an isopeptide bond (red) to SpyCatcher003. Ca^2+^ promotes aspartic anhydride formation, allowing reaction with the biotin-linked nucleophile (lilac). Anchored biotin is detected using streptavidin-HRP (green). **b** Structure of biotin nucleophiles. **c** Nucleophile reactivity with anhydride. Nucleophiles at 0.1, 1, or 10 mM were incubated at 37 °C for 30 min in HEPES buffer pH 7.4 with Ca^2+^ and conjugation was determined colorimetrically (mean and individual data points, *n* = 3). The buffer control consisted of 10% (v/v) DMSO in buffer in place of the nucleophiles. Source data are provided as a Source Data file.

In the OAZ/ODC model system, we established coupling via NeissLock to the α-amine or ε-amines on ODC. To further understand the reactivity of the SPM-generated aspartic anhydride, we developed a plate-based assay to compare a panel of biotin-linked nucleophiles, including those mimicking cysteine, tyrosine, α-amine, and ε-amine (Fig. 5a, b). SDS-PAGE is slow and usually involves sample heating; this assay was designed to be mild to facilitate detection of adducts of limited stability such as thioesters. SpyTag-Y-SPM was immobilized via covalent reaction with SpyCatcher003-sfGFP coated on a microplate. Varying concentrations of biotinylated nucleophiles were then incubated with SpyTag-Y-SPM in the presence of calcium. Coupled biotin was detected by addition of streptavidin labeled with horse radish peroxidase (HRP) (Fig. 5a). The thiol group in biotin-PEG-SH showed the highest reactivity. There was efficient reaction at the intermediate 1 mM concentration also by the α-amino group in biocytinamide (Fig. 5c). The ε-amino group in biotin pentyldiamine was found to be less reactive than the α-amine and its reactivity was similar to the phenol group in biotin tyramide (Fig. 5c). Therefore, this assay suggests that NeissLock could potentially crosslink to residues beyond lysine, such as cysteine or tyrosine residues in target proteins (forming more labile thioester or ester adducts).

### NeissLock coupling at the cell surface

**Fig. 6:**
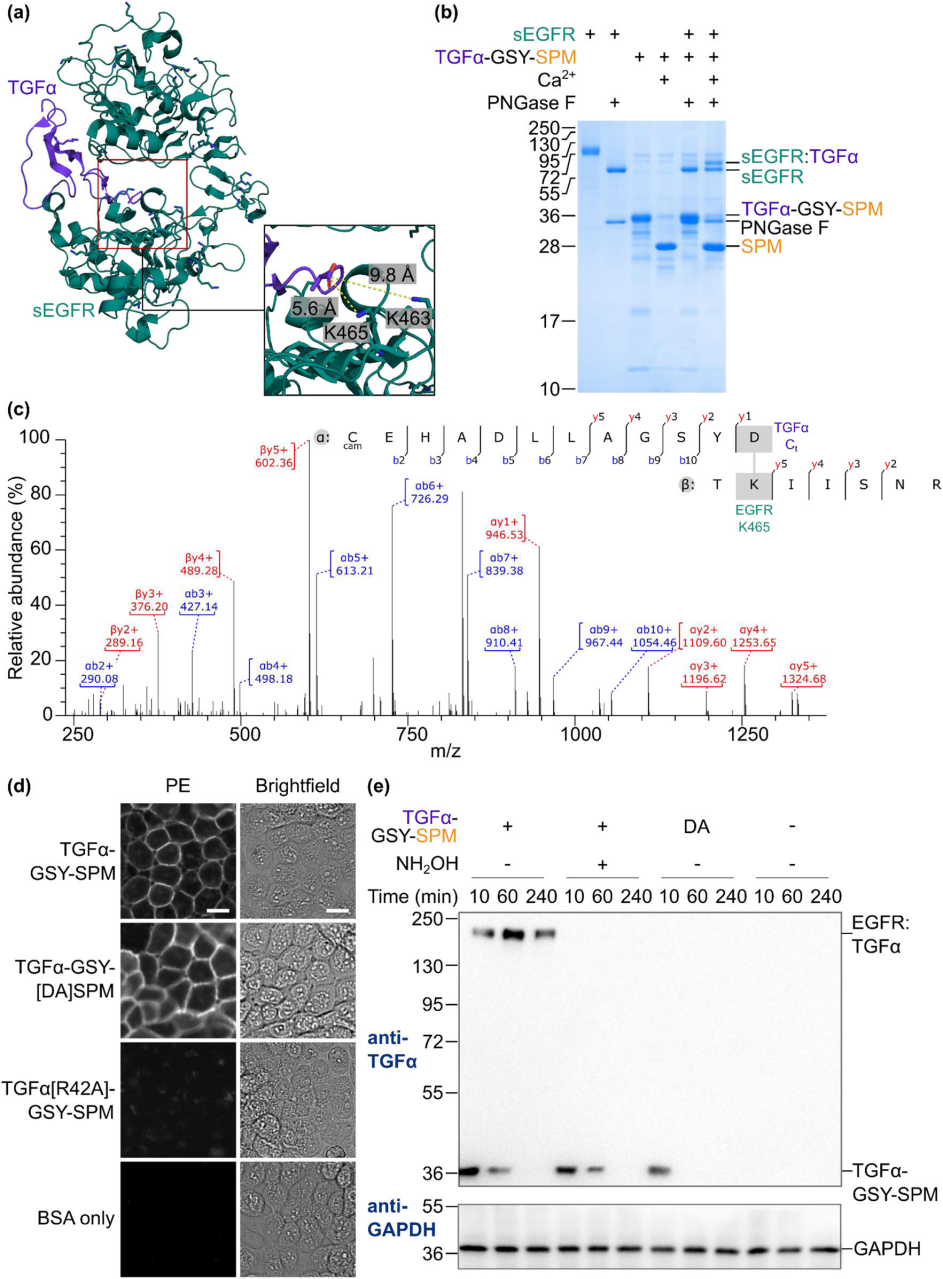
NeissLock for cellular labeling. **a** Crystal structure of complex between the extracellular region of EGFR (green) and TGFα (purple) (PDB 1mox). Inset shows the C-terminus of TGFa (chain C) and the distance to the nearest amines. **b** NeissLock reaction with soluble EGFR. TGFα-GSY-SPM was incubated with sEGFR ± Ca^2+^ for 5 h at 37 °C, before SDS-PAGE with Coomassie staining. PNGase F deglycosylation simplified sEGFR mobility. **c** MS mapping of the crosslink. Tryptic LC–MS/MS from reaction of TGFα-GSY-SPM with sEGFR identified a crosslink at K465. cam = carbamidomethylated. **d** TGFα-GSY-SPM retained specific binding to cell-surface EGFR. A431 cells were incubated with TGFα-GSY-SPM, non-reactive TGFα-GSY-[DA]SPM or reduced-binding TGFα[R42A]-GSY-SPM each at 5 µM or with BSA buffer control, before wide-field fluorescence microscopy. Brightfield and PE (detecting His-tag) fluorescence images are shown. Scale bar 20 µm. **e** NeissLock conjugation was specific to EGFR on cells and was abolished by addition of hydroxylamine. A431 cells were labeled for 10 min with 1 µM TGFα-GSY-SPM or non-reactive TGFα-GSY-[DA]SPM with 2 mM Ca^2+^ and ± hydroxylamine. Cells were incubated for the indicated time and blotted against TGFα. GAPDH was the sample processing control. Molecular weight markers represent kDa. Source data are provided as a Source Data file.

After proof-of-concept using the OAZ/ODC model system, we wanted to establish NeissLock for coupling of an important target at the mammalian cell surface. Based on NeissDist, we identified the complex between Transforming Growth Factor-alpha (TGFα) and Epidermal Growth Factor Receptor (EGFR) as a promising candidate (Fig. 6a, Supplementary Table 1). TGFα/EGFR signal activation has diverse effects in the body, including gastric acid secretion and release of mucus, but it is best studied for its role in cell proliferation and cancer^31^. First, we validated that this complex was suitable by testing conjugation of TGFα-GSY-SPM to the soluble ectodomain of EGFR (sEGFR)^32^ in vitro. Glycosylation of sEGFR in Expi293 cells led to heterogeneous gel mobility. Therefore, we expressed sEGFR in the presence of the mannosidase inhibitor kifunensine and deglycosylated sEGFR with Peptide:N-glycosidase (PNGase) F, resulting in a single sharp band for sEGFR (Fig. 6b). Incubation of sEGFR with TGFα-GSY-SPM in the presence of Ca^2+^ led to the formation of a new species, a covalent complex between sEGFR and TGFα, which is not present from autoproteolysis of TGFα-GSY-SPM alone (Fig. 6b). Using tryptic LC-MS/MS, we identified the crosslink to TGFα at K465 of sEGFR (Fig. 6c). This reaction is consistent with the closest amine to TGFα’s C-terminus (Fig. 6a) and the prediction of NeissDist (Supplementary Table 1).

SPM is a largely disordered domain, which becomes well-structured only upon binding of Ca^2+^ ^18–20^. To test that SPM does not promote promiscuous cellular interaction, we tested the binding of TGFα-GSY-SPM at the mammalian cell surface. Here, we used A431, a human epidermoid carcinoma cell-line expressing EGFR^33^. TGFα-GSY-SPM bound strongly to the cell-surface, as did the non-reactive TGFα-GSY-[DA]SPM control (Fig. 6d). However, with R42A point mutation in TGFα, known to interfere with EGFR binding^34^, the SPM fusion gave minimal cell staining (Fig. 6d).

We then tested NeissLock reaction of TGFα-GSY-SPM with EGFR on cells. A431 cells were labeled with TGFα-GSY-SPM in the presence of Ca^2+^, incubated for various times, and blotted against TGFα. Initially we detected that cells had unconjugated TGFα-GSY-SPM, as well as a high M_w_ form consistent with covalent conjugation of TGFα to EGFR (Fig. 6e). At 60 min and 240 min, the covalent EGFR:TGFα conjugate dominated, indicating efficient covalent coupling on cells (Fig. 6e). We found minimal bands from TGFα reaction with other proteins on the cell (Fig. 6e), consistent with high specificity of this NeissLock probe. Excess of the competing strong nucleophile hydroxylamine or the DA point mutation abolished the formation of this EGFR:TGFα adduct (Fig. 6e). We also observed decreasing abundance of EGFR:TGFα conjugate over time, indicating EGFR:TGFα degradation by the cell (Fig. 6e). We extended this time-course out to 5 h, while simultaneously monitoring total EGFR levels in these cells (Supplementary Fig. 9a). To investigate possible changes in signaling from covalent conjugation to EGFR, we probed levels of phosphorylated Signal Transducer and Activator of Transcription 1 (pSTAT1), a transcription factor and signaling effector downstream of EGFR^35^. We found that covalent conjugation to EGFR using TGFα-GSY-SPM led to increased pSTAT1 activation at 60 and 240 min, compared to the TGFα-GSY-[DA]SPM control or the hydroxylamine control (blocking covalent coupling) (Supplementary Fig. 9b). These results suggest that covalent conjugation of TGFα to EGFR may lead to more sustained pSTAT1 signaling by the cells.

## Discussion

Here we have established a rational approach for covalent targeting of endogenous proteins based on the standard genetic code, using chemistry that is inducible by mild cell-friendly conditions. We validated NeissLock reaction on three protein targets- ODC, AZI, and EGFR. To gain insight into the SPM system and the inconsistent efficiency of fusion constructs, we showed that the residue preceding the aspartate-proline scissile bond was key to reaction speed. The broad range of cleavage rates seen by changing this preceding residue should enhance the use of SPM for purification applications, as well as informing the rational design of slow-acting and fast-acting covalent probes for NeissLock.

While surveying the PDB, we anticipated that strict distance requirements would need to be satisfied between the reacting nucleophile and anhydride. Previous studies of electrophilic probes, either based on small-molecules or proteins, emphasize the importance of precise nucleophile positioning^9,11,36^. However, through exploration of the ODC/OAZ model complex, we observed a high tolerance for nucleophile position. When the most proximal amine (K92) was mutated out, K121 at 14 Å from OAZ’s C-terminal E219 still reacted efficiently. Therefore, given the abundance of lysines on protein surfaces, many protein complexes should have a suitable lysine in range, particularly when including a GSY-linker before SPM. In fact, we found thousands of such instances, promising broad applicability (Supplementary Data 1). On the other hand, this distance-tolerance complicates the assignment of crosslinks by MS and a larger area of the binder protein surface near the C-terminus may need to be free of amines to minimize self-reaction. If nearby lysines are present on the surface of the binding protein, self-conjugation could often be reduced by site-directed mutagenesis.

Apart from distance constraints, we were concerned that many ε-amines would be well-positioned for attack but have a pK_a_ only allowing reaction at pH 9-10. Instead, we found efficient conjugation even at pH 6.5–7.5. Our data show that the α-amine can be effective at conjugation, giving high coupling yield especially when K92 was unavailable. With a typical pK_a_ of 7.7^27^, the α-amine could therefore provide a compensatory effect at pH 6.5 or 7.0. However, conjugation to K92 or K121 was efficient at pH 7.4 even after α-amine reactivity was prevented. Hyperactivation of lysine in protein microenvironments can drive specific conjugation to electrophilic small-molecules^37^. No unusual pK_a_ values for each lysine in ODC were predicted by modeling using Rosetta (Supplementary Table 2)^38^. In a systematic assessment of lysine reactivity with electrophiles, there was poor correlation between predicted pK_a_; instead, the degree of surface exposure and local electrostatic interactions were key^39^. It is likely that the life-time of the anhydride before hydrolysis allows exploration of a substantial area of the target protein, particularly if the anhydride is connected by a flexible linker, so that even transient formation of the reactive -NH_2_ should allow reaction. Unlike more hydrolysis-resistant attenuated electrophiles used for unnatural amino acids^4^, the heightened reactivity of the anhydride electrophile could therefore permit efficient conjugation at physiological pH. We found that biotin-linked thiol could react with the aspartic anhydride. However, since lysine is much more common than cysteine at the protein surface and thioesters have limited stability, the focus of our study was on lysine positioning and reactivity.

Previous approaches for proximity ligation have either used UV induction of highly reactive free radicals or weak electrophiles^4^. UV-induced photocrosslinking is excellent for research applications but faces challenges because of DNA-damaging phototoxicity and limited tissue-penetration of UV light^40^. The use of constitutive weak electrophiles for proximity ligation of proteins is a precarious balancing act between too low reactivity (leading to slow reaction) or too high reactivity (leading to non-specific coupling and spontaneous inactivation upon storage)^4,41^. NeissLock gives a system with intrinsic low reactivity (normal amino acid side-chains) until high reactivity is induced by the mild conditions of typical extracellular calcium concentration. Since anhydrides have high reactivity, they have been little explored for directed protein modification. In neutral conditions, succinic anhydride or glutaric anhydride hydrolyzes with t_½_ ~4 min at 25 °C^42^. Compared to constitutive electrophiles, the anhydride is generated in situ, offering high reactivity to allow efficient coupling, although raising concerns about non-specificity. We found minimal non-specific reaction of OAZ-GSY-SPM with irrelevant non-binding proteins. Fine tuning of reactivity could include modifying accessibility of the reactive C-terminus, change of pH, removal/addition of competing self-reacting residues, or addition of free nucleophile to modulate anhydride life-time.

We demonstrated NeissLock conjugation of TGFα to EGFR on live cells. We observed changes in STAT1 signaling post conjugation of TGFα to EGFR. More study will be required to further understand the changes in signaling pathways from covalent coupling to cell surface receptors. EGFR can be rapidly internalized into acidic compartments upon ligand binding^43^, so future work will investigate NeissLock in different cellular locations. Since even peptide tags of 6 residues can induce immune responses^44^, it is important that the protein retains minimal foreign sequence for potential use in vivo. NeissLock leaves a small scar, i.e., just D, although a short spacer may improve performance. In summary, NeissLock should allow generation of covalent probes for a broad range of protein assemblies, increasing their resilience to time, force, or harsh conditions, with future potential for diagnostic and therapeutic applications^2,3,5,41^.

## Methods

### Plasmids and cloning

For cloning of constructs, Q5 High-Fidelity Polymerase (NEB) or KOD polymerase (EMD Millipore) was used for PCR, followed by Gibson assembly. Residue numbers for SPM derive from FrpC of *N. meningitidis* serogroup B (strain MC58) (UniProt Q9JYV5). The SPM sequence was based on residues 414–657 of FrpC^21^. When testing C-terminal truncations of SPM, SPM635 was based on residues 414–635, SPM613 on 414–613 and SPM591 on 414–591. pET28a-His_6_-SpyTag-A-SPM (GenBank MW364947) has the following organization: (M)GSS-linker, His_6_–tag, SSG-linker, thrombin cleavage site, NdeI restriction site, G-spacer, SpyTag, A, SPM, GSG-linker, C-tag. pET28a-His_6_-SpyTag-X-SPM has the alanine replaced by each of the other 19 amino acids. Residue numbers for OAZ and ODC were based on the crystal structure of the human OAZ/ODC complex (PDB 4zgy)^24^. pET28a-His_6_-OAZ-Y-SPM-Ctag has the following organization: N-terminal (M)GSS-linker, His_6_–tag, residues 95–219 of human OAZ1 (UniProt P54368), Y-spacer, SPM, GSG-linker, C-tag. pET28a-His_6_-OAZ-GSY-SPM-Ctag (GenBank MW364943, Addgene plasmid # 163613) has the same organization, except for the addition of a GS-spacer before Y. C175A was introduced into OAZ in pET28a-AviTag-His_6_-OAZ-GSY-SPM-Ctag by site-directed mutagenesis using Gibson assembly, to minimize any disulfide-mediated aggregation. The mutations K153A, K153E, V198A, A215R, and R188E were introduced into OAZ by site-directed mutagenesis using Gibson assembly to disrupt binding to ODC. D414A was introduced into SPM of OAZ-GSY-SPM by site-directed mutagenesis using Gibson assembly to generate a non-cleaving SPM variant. Human ODC1 (UniProt P11926) was cloned as pET28a-His_6_-ODC-Ctag (GenBank MW364944, Addgene plasmid #163614) to give the following organization: (M)GSS-linker, His_6_–tag, SSG-linker, ODC1, GSG-linker, C-tag. The ΔN version of ODC had the organization ODC1(10–460), GSG-linker, C-tag. The ΔN ΔC version of ODC had the organization ODC1(10–421) with K92R, K121R, K74R, and K78R mutations, GSG-linker, C-tag (GenBank MW364945). Human AZI1 (UniProt O14977) was cloned as pET28a-His_6_-AZI-Ctag (GenBank MW364946) to give the following organization: (M)GSS-linker, His_6_–tag, SSG-linker, AZI1, GSG-linker, C- tag. pET28a-TGFα-GSY-SPM (GenBank MW364948, Addgene plasmid #163615) includes mature TGFα sequence that was taken from residues 40–89 of human protransforming growth factor alpha (UniProt P01135). pET28a-TGFα-GSY-SPM has the following organization: (M), TGFα, GSY-linker, SPM, GSS-linker, His_6_-tag, SSG-linker, C-tag. DNA primers and gene fragments codon-optimized for *E. coli* expression were ordered from Integrated DNA Technologies before cloning into the pET28a backbone. pET28a-SpyTag003-sfGFP (Addgene plasmid #133454) and pET28a-SpyCatcher003-sfGFP (Addgene plasmid #133449) have been described^45^. pET28a-AviTag-DogTag-MBP has the following organization: (M)GSS-linker, His_6_–tag, SSG-linker, Thrombin site, AviTag, GSG-linker, DogTag, GSGESG-linker, MBP^46^. pENTR4-sEGFR-His_6_ has the organization: tissue plasminogen activator leader sequence, soluble fragment of extracellular domain of human EGFR (UniProt P00533, residues 25–525)^47^, GSGESG, His_6_. All constructs were validated by Sanger sequencing. pGEX-2T encoding GST-BirA was a gift from Chris O’Callaghan, University of Oxford, and purified by glutathione-affinity chromatography^48^. Oligonucleotides used in this study are listed in Supplementary Data 2.

### NeissDist – database search for model protein complex

To identify candidates for covalent fusion by C-terminal activation, protein structures were screened for the distance between the C-terminal resolved residue and the N-terminal resolved residue or lysine residues. Protein structures were retrieved from the worldwide protein data bank (wwPDB)^49^. Biological assemblies were generated using code provided by PDBx/mmCIF Dictionary Resources (mmcif.wwpdb.org/docs/sw-examples/python/html/assemblies.html). To preserve computational resources, the file-size of biological assemblies was limited prior to analysis to < 10 MB (e.g., to avoid analysis of viruses or similar large assemblies). For structures with multiple models, only the first 10 were analyzed. For each structure, initial analysis was performed using Python (Python Software Foundation) with the code we have provided (https://github.com/arnescheu/NeissDist). Herein, the Biopython PDB module was used to interpret structural data and parse dictionary information^50,51^. As parsed by the Biopython module, the last resolved standard amino acid for each polypeptide chain was defined as the ‘C-terminal’ residue for that chain. The first resolved standard amino acid residue in each polypeptide chain as well as all lysines were defined as ‘target’ residues. If applicable atoms were resolved in the structure, distances were calculated from atoms C (main-chain carbonyl carbon), CA (alpha carbon) and N (main-chain nitrogen) in the backbone of C-terminal residues to atoms CA and N in the backbone of the target residues as well as Nε in the side-chain of target lysines. For lysine, ‘primary distance’ was chosen by the first available distance in order of C-terminal to target residue: C to Nε > CA to Nε > N to Nε > C to CA > CA to CA > N to CA > C to N > CA to N > N to N. For N-terminal residues, ‘primary distance’ was chosen in order of C-terminal to target residue: C to αN > CA to αN > N to αN > C to CA > CA to CA > N to CA. If the N-terminal residue was also Lys, αN or Nε was selected, if available, according to which resulted in a shorter distance (i.e., C to αN/Nε > CA to αN/Nε > N to αN/Nε > C to CA > CA to CA > N to CA).

For Fig. 2c, d as well as Supplementary Data 1, we selected minimal distances using SQL grouped by category of distance (intramolecular, intermolecular homomeric, intermolecular heteromeric as assigned from NeissDist metadata). ‘Homomeric’ refers to distances between two identical protein sequences. ‘Heteromeric’ refers to distances between two non-identical protein sequences. Herein, only primary distances from C_t_ atom C to lysine atom Nε were considered (i.e., both C_t_ atom C and lysine atom Nε resolved, and C to Nε < C to αN for N-terminal lysines). For all biological assemblies generated from a single asymmetric unit, one distance was retrieved per category (which can be across assemblies). Using the SQLalchemy module in Python, this dataset was interrogated for all available structures of a given category in 0.1 Å intervals from 0 to 50 Å. Increments were binned into 1 Å intervals. In Fig. 2d, a single overall shortest distance was selected per structure, as an additional step before interrogation using SQLalchemy. Distances that were identified by NeissDist <1.6 Å represented covalent linkage, e.g., ubiquitination, and so were filtered out from Supplementary Data 1.

### SpyTag-X-SPM small-scale protein expression and purification

For pET28a-His_6_-SpyTag-X-SPM and related plasmids, plasmids were transformed into chemically-competent *E. coli* BL21 (DE3) RIPL (Agilent Technologies). Cells were plated on LB agar with 50 μg/mL kanamycin and 34 µg/mL chloramphenicol and incubated for 16 h at 37 °C. Single colonies were picked to inoculate 60 mL of Auto-induction medium LB (AIMLB0205, Formedium) with 50 μg/mL kanamycin and 34 µg/mL chloramphenicol in a 200 mL round-bottom flask. Cells were incubated for 24 h at 30 °C with 200 rpm shaking. Cells were harvested by centrifugation at 4000 g for 10 min at 4 °C. Cells were resuspended with 1.5 mL Ni-NTA buffer (50 mM Tris-HCl, 300 mM NaCl, pH 7.8) supplemented with cOmplete mini EDTA-free protease inhibitor cocktail (Roche), 1 mM phenylmethylsulfonyl fluoride (PMSF), 1 mg/mL lysozyme (Merck) and 2 U/mL benzonase and samples were transferred to microcentrifuge tubes. For lysis, cells were incubated at 25 °C with end-over-end rotation for 30 min and subjected to 6 rounds of freeze-thawing (−80 °C freezer to 23 °C thermomixer). After thawing, the total lysate was spun twice at 16,100–16,900 g for 30 min at 4 °C to separate the cleared lysate. Cleared lysate was incubated with 100 µL 1:1 slurry of pre-equilibrated Ni-NTA beads in Ni-NTA buffer at 4 °C before transfer to a 96-well filter plate (AcroPrep). Flow-through was collected by centrifugation at 300 g for 30 s. Samples were washed twice with 300 µL Ni-NTA binding buffer supplemented with 10 mM imidazole (each spin 10 s, 300 g, 4 °C) and then washed twice with 300 µL Ni-NTA buffer supplemented with 30 mM imidazole (each spin 10 s, 300 g, 4 °C). Finally, the protein was eluted with 300 µL Ni-NTA buffer supplemented with 200 mM imidazole. Eluates were concentrated using Vivaspin 500 5 kDa spin concentrators. The samples were buffer-exchanged into HEPES-buffered saline (HBS: 50 mM HEPES, 150 mM NaCl, pH 7.4) using the same concentrators. Samples were flash-frozen in an ethanol/dry ice bath and stored at −80 °C until further use.

### Other bacterial protein expression and purification

For pET28a-His_6_-OAZ-SPM-Ctag, pET28-His_6_-ODC1-Ctag, pET28-His_6_-AZI1-Ctag or related plasmids as well as pET28a-TGFα-GSY-SPM-Ctag, the plasmids were transformed into chemically-competent *E. coli* BL21 (DE3) RIPL. pET28a-TGFα-GSY-SPM-Ctag was transformed into *E. coli* BL21 (DE3) RIPL or *E. coli* T7 Express (NEB). Cells were then plated on LB agar with 50 μg/mL kanamycin and incubated for 16 h at 37 °C. Single colonies were picked to inoculate 11 mL of LB with 50 μg/mL kanamycin and 34 μg/mL chloramphenicol, before 16–19 h incubation at 37 °C with shaking at 200 rpm. 10 mL of the overnight culture was used to inoculate 1 L of LB with 50 μg/mL kanamycin and 34 μg/mL chloramphenicol in a baffled flask. Cultures were incubated at 37 °C with shaking at 200 rpm until A_600_ reached 0.6. Then the cultures were induced using 0.42 mM isopropyl β-D-1-thiogalactopyranoside (IPTG) (Fluorochem) and incubated at 25 °C with shaking at 200 rpm for 16–18 h. For constructs containing TGFα, induction was at 18 °C. Cell pellets were immediately processed or stored at −80 °C until further use.

Bacterial cells were harvested and lysed by sonication in lysis buffer [30 mM Tris-HCl, 200 mM NaCl, 5% (v/v) Glycerol, 15 mM imidazole, pH 7.5] supplemented with cOmplete mini EDTA-free protease inhibitor cocktail, 1 mM PMSF, 1 mg/mL lysozyme (Merck) and 2 U/mL benzonase (Merck). While kept on ice, the lysate was sonicated 3–4 times for 1 min at 50% duty cycle with 1 min rest periods. The cell lysate was then centrifuged in microcentrifuge tubes in a benchtop centrifuge at 16,900 g for 10–30 min at 4 °C or with a JA25−50 rotor (Beckman) at 30,000–35,000 g for 30–40 min at 4 °C.

For His_6_-tag purification, the clarified lysate was then added to pre-equilibrated Ni-NTA resin (Qiagen). After addition to a Poly-Prep gravity column, the Ni-NTA resin was washed with Ni-NTA buffer with 10 mM imidazole, followed by Ni-NTA buffer with 30 mM imidazole. The protein was eluted from the resin using Ni-NTA buffer with 200 mM imidazole. For variants of ODC, OAZ, and AZI, the lysis buffer, wash buffers and elution buffer were supplemented with 5 mM 2-mercaptoethanol.

For ODC variants without an N-terminal His-tag, the clarified lysate was added to CaptureSelect™ C-tagXL Affinity Matrix (Thermo Fisher). After addition to a Poly-Prep gravity column, the resin was washed with 20 mM Tris-HCl, 5 mM 2-mercaptoethanol, pH 7.4. The protein was eluted using 50 mM HEPES, 5 mM 2-mercaptoethanol, and 2 M MgCl_2_, pH 7.8.

After Ni-NTA or C-tag purification, variants of TGFα-GSY-SPM, ODC, OAZ-SPM, and AZI were concentrated using a Vivaspin centrifugal concentrator with 5, 10, or 30 kDa cut-off (GE Healthcare) and loaded onto a pre-equilibrated HiLoad 16/600 Superdex 200 pg size exclusion chromatography (SEC) column (GE Healthcare) connected to an ÄKTA Pure 25 (GE Healthcare) fast protein liquid chromatography (FPLC) machine at 4 °C. 50 mM HEPES, 150 mM NaCl pH 7.4 buffer was used for SEC of TGFα-GSY-SPM. SEC buffer was supplemented with 2 mM tris(2-carboxyethyl)phosphine (TCEP) for OAZ-SPM and AZI variants and with 2 mM TCEP as well as an additional 0.02 mM pyridoxal phosphate (PLP) as cofactor for ODC variants. Fractions were collected according to the A_280_ peak and verified by SDS-PAGE, before another round of concentration using pre-rinsed Vivaspin centrifugal concentrator with 5, 10, or 30 kDa cut-off (GE Healthcare) and then storage at −80 °C.

### Mammalian protein expression and purification

Expi293 cells were seeded in Expi293 Expression Medium (Thermo Fisher) at 2.5–3 × 10^6^ cells/mL in 25 mL volume. 80 µL ExpiFectamine (Thermo Fisher) in 1.4 mL Expi293 Expression Medium was incubated for 5 min and then combined with 25 µg of 0.22 µm filter-sterilized pENTR4-sEGFR-His_6_ diluted with 1.5 mL Expi293 Expression Medium. After 20 min incubation, the mixture was slowly added to the cell suspension. Where indicated, 30 µL of 5 mM kifunensine (Sigma-Aldrich) was added to the suspension. Cells were incubated at 37 °C, 8% (v/v) CO_2_ and 125 rpm orbital shaking. After 20 h, enhancer 1 (150 µL) and enhancer 2 (1.5 mL), as well as 150  µL of 100× Penicillin/Streptomycin (Gibco, for final 50 U/mL each), were added. Cells were grown for an additional 4 days, after which sEGFR was recovered from the cell supernatant using Ni-NTA affinity purification. Cell supernatant was supplemented with 5 mL Ni-NTA buffer to adjust pH, as well as mixed protease inhibitors (cOmplete mini EDTA-free protease inhibitor cocktail, Roche) and 1 mM PMSF. The supernatant was incubated with 5 mL of 1:10 Ni-NTA bead slurry (0.5 mL beads equilibrated to 5 mL in Ni-NTA buffer) at 4 °C with horizontal rolling at 33 rpm (Stuart roller mixer SRT6). Samples were applied to a gravity-flow column and washed twice with 10 mL Ni-NTA buffer supplemented with 10 mM imidazole and once with 2 mL Ni-NTA buffer supplemented with 30 mM imidazole. Samples were eluted with Ni-NTA buffer supplemented with 200 mM imidazole. Eluates were combined before buffer exchange and spin concentration using 30 kDa Vivaspin centrifugal concentrators. The purification yielded 0.21 mg sEGFR (weight estimated without considering glycosylation).

### Protein analysis

Protein concentrations were measured from A_280_, with extinction coefficients estimated using the ExPASy server^52^. SDS-PAGE employed 10%, 16%, or 18% polyacrylamide gels in an XCell SureLock system (Thermo Fisher) run at 180 V or 200 V. Optimized conditions for OAZ-Y-SPM or OAZ-GSY-SPM conjugation to ODC refers to 18% SDS-PAGE at 180 V for 110 min. Gels were stained using InstantBlue (Expedeon) and destained with MilliQ water before imaging with a ChemiDoc XRS imager. Quantification was carried out using Image Lab software (Bio-Rad, version 5.2.1 or 6.0.1)

### SPM cleavage and protein conjugation assays

Reactions were carried out at 37 °C in HBS supplemented with 2 mM TCEP. When measuring pH-dependence, an additional 50 mM 2-(N-morpholino)ethanesulfonic acid (MES) was added for proper buffering over the pH range tested. For reactions analyzing the effect of the −1 position on cleavage rate, 4–6 μM SpyTag-X-SPM was used with 10 mM cysteine in 50 mM HEPES, 150 mM NaCl, pH 7.4. For reactions analyzing the speed and pH-dependence of coupling, OAZ-SPM was reacted with ODC with each protein at 10 μM or at the indicated concentrations. When investigating buffer-dependence, TBS consisted of 20 mM Tris-HCl, 150 mM NaCl, pH 7.4 supplemented with 2 mM TCEP. OAZ-GSY-SPM was reacted with AZI with each protein at 2.5 μM for both the overnight reaction as well as the time course of conjugation. The cleavage of SPM was induced by addition of the HEPES reaction buffer, pre-equilibrated to 37 °C, containing calcium chloride at a final 10 mM. When investigating the calcium-dependence of the reaction, calcium chloride diluted in HEPES reaction buffer was added to give final concentrations of 2 mM, 1 mM or 0.5 mM calcium chloride. After the indicated time, the reaction was stopped by addition of 5× SDS-loading buffer [0.19 M Tris-HCl pH 6.8, 20% (v/v) glycerol, 100 μM bromophenol blue, 0.19 M SDS] containing EDTA added for a final 15 mM. The reaction was stopped with only EDTA to allow subsequent MS. SDS-loading buffer was added only for subsequent SDS-PAGE. Protein samples were then heated on a Bio-Rad C1000 thermal cycler at 95 °C for 6 min or 99 °C for 3 min. For time-courses, the 0 h time point was taken by addition of the stop buffer to the reaction before addition of the start buffer. Replicates represent multiple measurements derived from the same protein preparation. The fraction of uncleaved SPM was determined by dividing the intensity of the band for SpyTag-X-SPM or OAZ-SPM at each time point by the intensity of the SpyTag-X-SPM or OAZ-SPM band at the 0 h time point, i.e.,:

1 $${\mathrm{\% }}_{{\mathrm{uncleaved}}}\left( {\mathrm{t}} \right) = \frac{{{\mathrm{intensity}}_{{\mathrm{uncleaved}}}\left( {\mathrm{t}} \right)}}{{{\mathrm{intensity}}_{{\mathrm{uncleaved}}}\left( 0 \right)}} \times 100$$

The fraction of conjugated ODC was determined by dividing the decrease in intensity of the band for ODC by the intensity of the ODC band at the 0 h time point, i.e.,:

2 $${\mathrm{\% }}_{{\mathrm{conjugated}}}\left( {\mathrm{t}} \right) = \frac{{{\mathrm{intensity}}_{{\mathrm{ODC}}}\left( 0 \right) - {\mathrm{intensity}}_{{\mathrm{ODC}}}\left( {\mathrm{t}} \right)}}{{{\mathrm{intensity}}_{{\mathrm{ODC}}}\left( 0 \right)}} \times 100$$

The fraction of conjugated AZI was determined by dividing the decrease in intensity of the band for AZI by the intensity of the AZI band at the 0 h time point, i.e.,:

3 $${\mathrm{\% }}_{{\mathrm{conjugated}}}\left( {\mathrm{t}} \right) = \frac{{{\mathrm{intensity}}_{{\mathrm{AZI}}}\left( 0 \right) - {\mathrm{intensity}}_{{\mathrm{AZI}}}\left( {\mathrm{t}} \right)}}{{{\mathrm{intensity}}_{{\mathrm{AZI}}}\left( 0 \right)}} \times 100$$

The relative conjugation to ODC in the pH/buffer/calcium-dependence time-course was determined by dividing the intensity of the band for OAZ:ODC by the intensity of the OAZ:ODC band after 2 h for the most efficient reaction, i.e.,:

4 $${\mathrm{Relative}}\,{\mathrm{\% }}_{{\mathrm{conjugated}}}\left( {\mathrm{t}} \right) = \frac{{{\mathrm{intensity}}_{{\mathrm{OAZ}}:{\mathrm{ODC}}}\left( {\mathrm{t}} \right)}}{{{\mathrm{intensity}}_{{\mathrm{OAZ}}:{\mathrm{ODC}}}\left( {2{\mathrm{h}}} \right)}} \times 100$$

The relative conjugation to MBP compared to ODC was determined by dividing the intensity of the band for OAZ:MBP by the intensity of the OAZ:ODC band (adjusted for the relative molecular weights of OAZ:MBP and OAZ:ODC), i.e.,:

5 $${\mathrm{Relative}}\,{\mathrm{\% }}_{{\mathrm{conjugated}}}\left( {\mathrm{t}} \right)\,{\mathrm{ = }}\,\frac{{{\mathrm{intensity}}_{{\mathrm{OAZ:MBP}}}\left( {\mathrm{t}} \right)}}{{{\mathrm{intensity}}_{{\mathrm{OAZ:ODC}}}\left( {\mathrm{t}} \right)}}{\mathrm{ \times }}\frac{{{\mathrm{molecular}}\,{\mathrm{weight}}_{{\mathrm{OAZ:ODC}}}}}{{{\mathrm{molecular}}\,{\mathrm{weight}}_{{\mathrm{OAZ:MBP}}}}} \times {\mathrm{100}}$$

The relative conjugation to each ODC variant was determined, for example, by dividing the intensity of the band for OAZ: ΔN 4KR ODC by the intensity of the OAZ:ODC (wt) band (adjusted for the relative molecular weights of OAZ: ΔN 4KR ODC and OAZ:ODC (wt), i.e.,

6 $${\mathrm{Relative}}\,{\mathrm{\% }}_{{\mathrm{conjugated}}}\left( {\mathrm{t}} \right) = \frac{{{\mathrm{intensity}}_{{\Delta}{\mathrm{N}}{\,}4{\mathrm{KR}}\;{\mathrm{ODC}}}\left( {\mathrm{t}} \right)}}{{{\mathrm{intensity}}_{{\mathrm{OAZ}}:\,{\mathrm{ODC}}({\mathrm{wt}})}\left( {\mathrm{t}} \right)}} \times \frac{{{\mathrm{molecular}}\;{\mathrm{weight}}_{{\mathrm{OAZ:ODC}}({\mathrm{wt}})}}}{{{\mathrm{molecular}}\;{\mathrm{weight}}_{{\Delta}{\mathrm{N}}4{\mathrm{KR}}\;{\mathrm{ODC}}}}} \times 100$$

### OAZ coupling in cell lysate

A431 cell lysate was prepared by first washing A431 cells once with PBS before resuspending 2 × 10^7^ cells in 1 mL lysis buffer consisting of 20 mM Tris-HCl, 150 mM NaCl, 1% (v/v) Triton-X-100, pH 7.4, supplemented with mixed protease inhibitors and 1 mM PMSF. The cell lysate was incubated on ice for 20 min before centrifugation at 12,000 g for 10 min at 4 °C. 1 μM biotinylated AviTag-OAZ-GSY-SPM was added to the clear lysate, with or without 1 μM ODC. Cleavage of SPM was induced by addition of clear lysate containing calcium chloride at a final concentration of 2 mM. The reaction was carried out at 25 °C for 10 min, before the reaction was stopped by addition of 5× SDS-loading buffer containing EDTA at a final concentration of 15 mM. Samples were heated at 99 °C for 3 min before resolving by SDS-PAGE. Protein samples were transferred to a nitrocellulose membrane at 25 V for 10 min using the iBlot2 Dry Blotting System (Thermo Fisher). The membrane was blocked for 1 h at 25 °C with 5% (w/v) bovine serum albumin (BSA) in PBS with 0.05% (v/v) Tween-20 (Merck) (PBS-T) before incubation for 20 h at 4 °C with mouse anti-His (Invitrogen, HIS.H8) at 1:1,000 dilution in 5% (w/v) BSA in PBS-T. The membrane was then washed 6 times with PBS-T. Subsequently, the membrane was incubated with goat anti-mouse horseradish peroxidase (HRP) (A4416, Merck) at 1:1,000 in 5% (w/v) BSA in PBS-T for 1 h at 25 °C. The membrane was washed 6 times with PBS-T and incubated with SuperSignal West Pico PLUS Chemiluminescent Substrate. Chemiluminescence was measured using ChemiDoc XRS imager using Image Lab Software version 5.2.1 (Bio-Rad).

### SEC-MALS

OAZ-GSY-SPM at 2 mg/mL in 100 μL 50 mM HEPES, 150 mM NaCl, 2 mM TCEP, 0.02 mM PLP, pH 7.4 was injected at 25 °C into a Superdex 200 HR 10/30 column (GE Healthcare), connected to a Shimadzu HPLC system with a Wyatt Dawn HELEOS-II 8-angle light scattering detector and Wyatt Optilab rEX refractive index monitor. The running buffer was HBS with 2 mM TCEP and 0.02 mM PLP. ASTRA 6 software (Wyatt Technology) was used for analysis of the light scattering, refractive index, and UV absorbance data. Error bars in the observed molecular weight represent the uncertainty in fitting to the molar mass curve.

### Surface plasmon resonance

SPR was carried out using a Biacore T200 (GE Healthcare). AviTag-OAZ-GSY-SPM, AviTag-OAZ(K153A,V198A)-GSY-SPM, AviTag-OAZ(K153A,A215R)-GSY-SPM and AviTag-OAZ(K153E,R188E,V198A)-GSY-SPM were biotinylated using GST-BirA^48^. 50 µM of each AviTagged protein was incubated with 5 mM MgCl_2_, 1 mM ATP, 0.75 mM biotin, and 3.3 µM GST-BirA in PBS for 1 h at 25 °C. The same amount of GST-BirA was added again, before incubation for another for 1 h at 25 °C. GST-BirA was removed by incubation with Glutathione HiCap matrix (Qiagen) for 1 h at 4 °C, before spinning the resin down. Excess biotin was removed subsequently by gel filtration using the HiLoad 16/600 Superdex 200 pg size exclusion chromatography (SEC) column (GE Healthcare) into HBS with 2 mM TCEP. Biotinylated AviTag-OAZ-GSY-SPM, AviTag-OAZ(K153A,V198A)-GSY-SPM, AviTag-OAZ(K153A,A215R)-GSY-SPM or AviTag-OAZ(K153E,R188E,V198A)-GSY-SPM was immobilized onto the sensor chip using the Biotin CAPture Kit, Series S (GE Healthcare). C175A was introduced into OAZ to minimize any disulfide-mediated aggregation. The flow buffer was HBS with 2 mM TCEP and 0.02 mM PLP. Measurements were made at 25 °C. Following each run, the chip was regenerated following the manufacturer’s protocol. Biacore T200 Software v3.0 (GE Healthcare) was used for data analysis and a 1:1 binding model between OAZ and ODC was used for fitting and to derive the kinetic binding constants. Replicates represent multiple measurements derived from the same protein preparation.

### Mass spectrometry

For intact protein MS, we used a RapidFire 365 platform (Agilent) comprising a jet-stream electrospray ionization source coupled to a 6550 Accurate-Mass Quadrupole Time-of-Flight (Q-TOF) (Agilent) detector. 30 µM ODC was incubated with 10 µM OAZ-Y-SPM and 10 mM CaCl_2_ for 16 h at 37 °C in HBS with 2 mM TCEP. Processing was stopped by addition of EDTA to 15 mM (1/5 volume). Samples were diluted 1:1 with water and acidified to 0.9% (v/v) formic acid before aspiration under vacuum for 0.3 s and loading onto a C4 solid-phase extraction cartridge. Washes using 0.1% (v/v) formic acid in water were carried out for 5.5 s, before the sample was eluted onto the Q-TOF detector for 5.5 s. Data were analyzed using MassHunter Qualitative Analysis B.07.00 (Agilent), with the following deconvolution settings: Deconvolute (protein), maximum entropy deconvolution algorithm, mass range 10,000.00–80,000.00 Da, mass step 1,0000 Da, m/z range limited to 600,0000–5,000,0000 m/z, with baseline subtraction at baseline factor 3.00, proton adduct and automatic isotope width. Expected M_w_ was calculated using the ExPASy ProtParam tool. For ODC and OAZ-Y-SPM, we considered full-length protein with fMet removed. For post-cleavage OAZ-Y-D or SPM, the expected molecular weight of a corresponding protein was considered (i.e., no fMet, hydrolyzed anhydride for OAZ-Y-D). For the expected molecular weight of an ODC:OAZ-Y-D conjugate, masses for each protein were added and the mass of water (−18.01 Da) was subtracted.

### Tryptic LC–MS/MS

Conjugated protein samples were resolved on SDS-PAGE to separate different conjugate species. Bands were cut from the gel. For in-gel tryptic digest, gel slices were fragmented and transferred to LoBind tubes (Eppendorf). Gel pieces were covered with 50% (v/v) acetonitrile (ACN) + 50% (v/v) 100 mM ammonium bicarbonate and destained at 37 °C. Supernatant was removed and samples were reduced with 10 mM TCEP in 100 mM ammonium bicarbonate for 30 min at 25 °C, before drying the gel slices with ACN. Supernatant was removed and samples were incubated for 30 min at 25 °C with 50 mM 2-chloroacetamide in 100 mM ammonium bicarbonate in the dark (leading to carbamidomethylation of cysteines). Supernatant was removed, gel slices were washed twice with ACN and rehydrated with Sequencing Grade Modified Trypsin (Promega, reconstituted with 50 mM acetic acid) at 100 ng trypsin per band. After overnight digestion, supernatant was collected in LoBind tubes and tryptic digestion inhibited upon wash of gel pieces with 10% (v/v) formic acid. The supernatant was combined with the previous supernatant. Finally, remaining (crosslinked) peptides were extracted from the gel pieces with ACN. After transfer to a new LoBind tube, the solvent was removed by vacuum evaporation. Extracted peptides were resuspended with 5% (v/v) formic acid and 5% (v/v) dimethyl sulfoxide (DMSO) in water and combined with the previous supernatant. Samples were stored at 4 °C or frozen at −20 °C until analysis.

Peptides were separated on an EASY-nLC^53^ 1000 ultra-high-performance liquid chromatography (UHPLC) system (Proxeon) and electrosprayed directly into a Q Exactive mass spectrometer (Thermo Fisher). Peptides were trapped on a C18 PepMap100 pre-column (300 µm inner diameter × 5 mm, 100 Å pore size, Thermo Fisher) using solvent A [0.1% (v/v) formic acid in water] at 500 bar and then separated on an in-house packed analytical column (50 cm × 75 µm inner diameter packed with ReproSil-Pur 120 C18-AQ, 1.9 µm, 120 Å pore size, Dr. Maisch GmbH) with a linear gradient from 10 to 55% (v/v) solvent B [0.1% (v/v) formic acid in ACN] in 45 min at 200 nL/min. Full scan MS spectra were acquired in the Orbitrap (scan range 350–2000 m/z, resolution 70,000, Automatic Gain Control target 3e6, maximum injection time 100 ms). After the MS scans, the 10 most intense peaks were selected for Higher-energy collisional dissociation (HCD) fragmentation at 30% of normalized collision energy. HCD spectra were also acquired in the Orbitrap (resolution 17,500, Automatic Gain Control target 5e4, maximum injection time 120 ms) with first fixed mass at 100 m/z. Charge states 1+ and 2+ were excluded from HCD fragmentation.

MS data were converted into Mascot generic format (mgf) using pParse and searched using the pLink software^54^. The database contained the target proteins and common contaminants. Search parameters were as follows: maximum number of missed cleavages = 2, fixed modification = carbamidomethyl-Cys, variable modification 1 = Oxidation-Met, variable modification 2 = Glu to pyro-Glu. Crosslinking from D to K, S, T, or N-terminus was considered. Data were initially filtered to a False-discovery rate (FDR) of 1%. Crosslinks were further filtered/inspected with specific emphasis on fragmentation patterns. Data were deposited in the ProteomeXchange Consortium via the PRIDE^55^ partner repository with the dataset identifier PXD023073.

### Plate-based assay of aspartic anhydride reaction with nucleophiles

A Nunc MaxiSorp 96-well flat-bottom ELISA plate (Thermo Fisher) was coated with 100 µL per well of SpyCatcher003-sfGFP at 2 µg/mL in PBS pH 7.4 (P4417, Merck) and incubated at 4 °C for 16–18 h. Excess unbound SpyCatcher003-sfGFP was removed by washing the plate 6 times with 400 µL PBS-T each time. Blocking was done by incubating each well with 200 µL 3% (w/v) BSA in PBS-T at 25 °C for 1 h. After another wash in PBS-T, 100 µL SpyTag-Y-SPM at 2 µg/ml in HBS was added to each well and incubated at 25 °C for 30 min. Unbound SpyTag-Y-SPM was removed by washing the plate 6 times with 400 µL PBS-T. Biotin pentyldiamine (10404034, Thermo Fisher), biocytinamide (Merck), biotin-PEG-alkoxyamine (26137, Thermo Fisher), biotin tyramide (Merck) and biotin-PEG-SH (M_w_ 3,000 Da) (compound PEG1213, Iris Biotech) were added to each well at varying final concentrations, dissolved in 10% (v/v) DMSO in HBS with 10 mM CaCl_2_. The buffer control consisted of 10% (v/v) DMSO in HBS with 10 mM CaCl_2_. The biotinylated ligands were incubated at 37 °C for 0.5 h, shaking at 300 rpm. Excess unbound ligands were removed by washing the plate 6 times with 400 µL PBS-T. 100 µL Pierce High Sensitivity Streptavidin-HRP (Thermo Fisher) dissolved 1:5,000 in 3% (w/v) BSA in PBS-T was added to each well and incubated at 25 °C for 1 h. The ELISA plate was washed 6 times with 400 µL PBS-T. 100 µL 1-Step Ultra 3,3’,5,5’-Tetramethylbenzidine (TMB) ELISA substrate (Thermo Fisher) was added to each well for 1 min, before the reaction was stopped by addition of 100 µL 1 M HCl. Absorbance at 450 nm was read using the FLUOstar Omega microplate plate reader (BMG Labtech).

### Lyophilization

100 μL OAZ-GSY-SPM in 50 mM HEPES, 150 mM NaCl, 2 mM TCEP, pH 7.4 buffer was first stored in −80 °C for 16 h to freeze the sample. Freeze-drying was carried out using a benchTop 2 K freeze-dryer (VisTis) for 20 h at 0.14 mbar and −72.5 °C. The lyophilized OAZ-GSY-SPM was then reconstituted in 100 μL MilliQ water and centrifuged at 16,900 g for 10 min. After the removal of aggregates, the supernatant was used to test for coupling to ODC by SDS-PAGE with Coomassie staining.

### sEGFR conjugation with TGFα-GSY-SPM

2.5 µM sEGFR was incubated with 12.5 µM TGFα-GSY-SPM and 2 mM CaCl_2_ in 50 mM HEPES, 150 mM NaCl, pH 7.4 at 37 °C. After 5 h, samples were incubated with 1× Glycoprotein Denaturing Buffer (10× stock, NEB). Samples were heated for 10 min at 100 °C. Subsequently, Glycoprotein Buffer 2 (NEB) was added to 1×, NP-10 (NEB) to 10% (v/v), and PNGase F (NEB) to 25,000 U/mL. Samples were digested for 1 h at 37 °C. Finally, SDS-loading buffer was added. Samples were heated to 95 °C for 6 min and resolved on SDS-PAGE, before Coomassie staining.

### Cell culture and fluorescence microscopy

A431 cells were from Cancer Research UK, Lincoln’s Inn Fields. Cells were cultured in complete media [Dulbecco’s Modified Eagle Medium–high glucose (DMEM) supplemented with 10% (v/v) fetal bovine serum (FBS), penicillin/streptomycin (Gibco, 100 U/mL), and 1× GlutaMAX (Gibco)] at 37 °C, with 5% (v/v) CO_2_. For cell staining, sub-confluent A431 were seeded at 2 × 10^4^ cells/cm^2^ onto glass-bottom petri dishes (MatTek). Cells were grown for 24 h, washed twice with serum-free medium (DMEM supplemented with 1× penicillin/1× streptomycin and 1× GlutaMAX) and then maintained for an additional 16–18 h at 37 °C with 5% (v/v) CO_2_ The dishes were transferred to 4 °C to prevent receptor internalization and cells were washed twice with cold 1 mL HBS-Mg (HBS with 5 mM MgCl_2_, pH 7.4). Cells were incubated at 4 °C with 5 µM TGFα-GSY-SPM (wt, DA or R42A versions) in HBS-Mg supplemented with 1% (w/v) BSA (A7906, Sigma) or only HBS-Mg with BSA as indicated. After 1 h, cells were washed twice with HBS-Mg. Subsequently, samples were incubated in the dark with 1 mL Anti-His-Phycoerythrin (BioLegend 362603) at 1:100 in HBS-Mg for 1 h at 4 °C. Cells were washed thrice and then covered with 1 mL HBS-Mg. Samples were imaged with a DeltaVision core inverted wide-field microscope with softWoRx software (Applied Precision and Micron Oxford), using brightfield or an mCherry filter for fluorescence imaging of PE (575/25 nm excitation, 625/45 nm emission). All fluorescence images were collected and analyzed using the same settings.

### Western blots

In total 5 × 10^5^ A431 cells per well were seeded into 6-well plates, grown at 37 °C with 5% (v/v) CO_2_ in complete media for 24 h, washed twice in 4 mL serum-free medium, and subsequently starved with serum-free medium for an additional 24 h at 37 °C with 5% (v/v) CO_2_. Prior to cell conjugation, cells were washed twice with 2 mL HBS-Mg. 150 µL TGFα-GSY-SPM (wt or DA) diluted to 2 µM in HBS-Mg with or without 5 mM hydroxylamine (Sigma-Aldrich) [final concentration 2% (v/v) DMSO] in HBS-Mg was added to cells. Rapidly thereafter, 150 µL HBS-Mg with 4 mM CaCl_2_ with or without 5 mM hydroxylamine [final concentration 2% (v/v) DMSO] was added to the cells, without prior removal of the protein solution. Cells were incubated for an additional 10 min, during which time they were placed in an incubator at 37 °C with 5% (v/v) CO_2_. The supernatant was removed and cells were incubated for the indicated time with serum-free medium with or without 5 mM hydroxylamine [final concentration 2% (v/v) DMSO] at 37 °C with 5% (v/v) CO_2_. Cells were washed twice with HBS-Mg. For cell lysis, 150 µL ice-cold modified RIPA buffer [20 mM Tris-HCl, 150 mM NaCl, 1 mM PMSF, 1% (v/v) Triton-X-100, 0.5% (w/v) sodium deoxycholate, 0.1% (w/v) SDS, 5 mM NaF, pH 7.4], supplemented with cOmplete mini EDTA-free protease inhibitor cocktail, 1 mM PMSF and 1 mM sodium orthovanadate was added to each well. Lysates were incubated on ice for 20 min, before centrifuging at 12,000 g at 4 °C for 20 min. The supernatant was mixed with 6× SDS loading buffer containing 120 mM dithiothreitol and heated for 6 min at 95 °C, before resolving on SDS-PAGE.

For Western blotting with anti-TGFα and anti-EGFR, proteins were transferred from SDS-PAGE to nitrocellulose membrane (Bio-Rad) in transfer buffer [7.2 g/L glycine, 1.44 g/L Tris base in 20% (v/v) methanol] for 16 h at 30 V at 4 °C. Membranes were blocked for 1 h at 25 °C with 5% (w/v) skim milk (Sigma-Aldrich) in PBS-T. Subsequently, membranes were incubated for 20 h at 4 °C with primary antibodies at 1:1,000 in 5% (w/v) skim milk in PBS-T, i.e., 0.2 µg/mL mouse anti-TGFα (MF9, Novus Biologicals) or mouse anti-EGFR (LA22, Sigma-Aldrich). Membranes were washed 6 times with PBS-T before addition of goat anti-mouse horseradish peroxidase (HRP) (A4416, Merck) at 1:1,000 in 5% (w/v) skim milk with PBS-T. After incubation at 25 °C for 1 h, membranes were washed 6 times with PBS-T.

For Western blotting with anti-pSTAT1 and anti-GAPDH (glyceraldehyde-3-phosphate dehydrogenase), proteins were transferred from SDS-PAGE to nitrocellulose (Bio-Rad) in transfer buffer for 3 h at 30 V at 25 °C. Membranes were blocked for 1 h at 25 °C with 5% (w/v) BSA (Sigma-Aldrich) in PBS-T. Subsequently, membranes were incubated for 20 h at 4 °C with primary antibodies at 1:1,000 in 5% (w/v) BSA in PBS-T, i.e., rabbit anti-pSTAT1(Y701) (58D6, Cell Signaling Technology) or mouse anti-GAPDH (GA1R, Thermo Fisher). Membranes were washed 6 times with PBS-T before addition of goat anti-rabbit horseradish peroxidase (HRP) (65–6120, Thermo Fisher) at 1:1,000 in 5% (w/v) BSA with PBS-T for anti-pSTAT1(Y701) or goat anti-mouse horseradish peroxidase (HRP) (A4416, Merck) at 1:1,000 in 5% (w/v) BSA with PBS-T for anti-GAPDH. After incubation at 25 °C for 1 h, membranes were washed 6 times with PBS-T. Membranes were incubated with SuperSignal West Pico PLUS Chemiluminescent Substrate before measuring chemiluminescence on a ChemiDoc XRS imager using Image Lab Software version 5.2.1 (Bio-Rad).

### Graphics and structure visualization

The structure of OAZ/ODC was obtained from PDB 4zgy^24^, OAZ/AZI from PDB 4zgz^24^, and TGFα/EGFR from PDB 1mox^47^. Structures were visualized using PyMOL version 2.2.0 (Schrödinger). Distances shown were drawn from atom C of the C-terminal resolved residue to atom Nε of lysine. Figures were prepared using the FIJI distribution of ImageJ^56^ and the open-source graphics editor Inkscape (inkscape.org). In Supplementary Fig. 1a, the secondary structure of SPM414-591 was derived from the NMR structure of FrpC SPM in PDB 6sjw^20^ and the secondary structure of SPM591-657 was predicted by the Jpred4 server^26^. FrpC414–657 was subject to Ginzu Domain Prediction using the Robetta server^25^. Figure 2b was designed using free icons made by Freepik, monkik, and itim2101 from www.flaticon.com. Chemical structures were prepared in ChemDraw.

pK_a_ values were predicted using the ROSIE Rosetta server^38^ based on the ODC/OAZ crystal structure (PDB 4zgy). Values were derived from a single run using the pK_a_ app with re-packing of neighbor residues at a pack radius of 6.0 Å and no pre-packing.

### Statistics and reproducibility

For kinetic analysis of protein conjugation or processing, replicate samples refer to multiple analyses from the same protein preparation, after which gels were processed and resolved in parallel. For representative SDS-PAGE (Fig. 3b, Fig. 4a, b, d, e, Fig. 6b, Supplementary Fig. 3, Supplementary Fig. 5b, Supplementary Fig. 6b), observations were confirmed at least once with similar or identical conditions. For Fig. 1e, proteins were processed in three sets of triplicates, before resolving and analyzing all samples in parallel. RapidFire-MS was confirmed at least once with similar conditions. For Fig. 6d, observations were confirmed once with similar conditions. For Fig. 6e, the experiment was repeated thrice independently with similar results. For Supplementary Fig. 4, K_d_ values are representative of two separate experiments. For Supplementary Fig. 5, the experiment was repeated twice independently with similar results. Regarding tryptic LC/MS-MS of OAZ-Y-SPM conjugation to wt ODC, similar findings were made with AviTag-OAZ-GSY-SPM. For SEC-MALS (Supplementary Fig. 7b), results were confirmed in the same experiment at a different protein dilution. For Supplementary Fig. 9a, the experiment was repeated twice independently with similar results. For Supplementary Fig. 9b, the experiment was repeated thrice independently with similar results.

### Reporting summary

Further information on research design is available in the Nature Research Reporting Summary linked to this article.

## Supplementary information

Supplementary Information

Description of Additional Supplementary Files

Supp Data 1

Supp Data 2

Reporting Summary

## Data Availability

Amino acid sequences are deposited in GenBank as described in the main text section Plasmids and cloning. Plasmids encoding OAZ-GSY-SPM, ODC, and TGFα-GSY-SPM were deposited in the Addgene repository (https://www.addgene.org/Mark_Howarth/). The mass spectrometry proteomics data have been deposited to the ProteomeXchange Consortium via the PRIDE^55^ partner repository with the dataset identifier PXD023073. Further information and request for resources and reagents should be directed to and will be fulfilled by the corresponding author. Source data are provided with this paper.

## Source data

Source data

## Code availability

Code used to generate and analyze the distance database is deposited on GitHub (https://github.com/arnescheu/NeissDist)^57^.
