# Supplementary material for: NeissLock provides an inducible protein anhydride for covalent targeting of endogenous proteins
Source: Nat Commun. 2021 Jan 29;12:717. doi: 10.1038/s41467-021-20963-5 (PMC7846742; doi:10.1038/s41467-021-20963-5)
Supplement: Supplementary file 1 — Supplementary Information [file 41467_2021_20963_MOESM1_ESM.pdf]

Supplementary Information

NeissLock provides an inducible protein anhydride for covalent targeting of endogenous proteins

Arne H. A. Scheu[1*], Sheryl Y. T. Lim[1*], Felix J. Metzner[1,2], Shabaz Mohammed[1], and Mark Howarth[1]†
[1]Department of Biochemistry, University of Oxford, South Parks Road, Oxford OX1 3QU, UK. [2]Current address: Gene Center and Department of Biochemistry, Ludwig-Maximilians-Universität München, Feodor-Lynen-Straße 25, 81377, Munich, Germany.
*Equal contribution †Corresponding author

PDB ID	Complex	Organism	Resolution (Å)	C-terminal residue	Target residue	Primary distance (Å)
1mox	Epidermal Growth Factor Receptor / Transforming Growth Factor alpha	*Homo sapiens*	2.5	Chain D (48 aa) A50	Chain B (501 aa) K465	3.3
4zgy	Ornithine Decarboxylase / Ornithine Decarboxylase Antizyme	*Homo sapiens*	2.6	Chain B (125 aa) E219	Chain A (383 aa) K92	3.5
1ory	Flagellar protein FliS / Flagellin	*Aquifex aeolicus*	2.4	Chain B (40 aa) R2518	Chain A (119 aa) K1028	3.8
2qac	Myosin A tail domain interacting protein MTIP / Myosin-A	*Plasmodium falciparum*	1.7	Chain A (144 aa) Q204	Chain T (14 aa) K813	3.9
1dml	DNA polymerase processivity factor / DNA polymerase	*Human herpesvirus 1*	2.7	Chain B (36 aa) A1235	Chain A (267 aa) K289	3.9
5yqz	Glucagon receptor / Endolysin / Glucagon analogue	*Homo sapiens, Enterobacteria phage T4*	3.0	Chain P (28 aa) T29	Chain R (558 aa) K64	4.1
1syx	Spliceosomal U5 snRNP-specific 15 kDa protein / CD2 antigen cytoplasmic tail-binding protein 2	*Homo sapiens*	2.3	Chain B (62 aa) T86	Chain A (135 aa) K125	4.3
1g0y	Interleukin-I receptor Type I / Antagonist peptide AF10847	*Homo sapiens*	3.0	Chain I (21 aa) L21	Chain R (310 aa) K95	5.5

Supplementary Table 1. Summary of example hits to illustrate NeissDist database. C-terminal residue: NeissDist selects the last resolved residue in a given polypeptide chain. Target residue: nearest lysine on the partner protein. In parentheses is the resolved chain length as annotated by NeissDist. Primary distance: distance from carbonyl carbon to Nε (see methods for further definition).

Residue	Predicted pK_a
K21	10.2
K27	9.8
K37	10.5
K50	10.4
K51	11.2
K57	10.4
K69	10.0
K74	10.3
K78	10.2
K92	11.1
K115	9.3
K121	10.3
K141	11.3
K148	10.5
K150	11.0
K169	11.8
K185	10.3
K245	10.4
K247	10.6
K261	11.4
K293	10.2
K294	10.2
K298	10.2
K337	10.4
K342	10.4
K349	10.3

Supplementary Table 2. Predicted pK_a values of lysine side-chains in ODC. Values were determined by Rosetta based on the ODC/OAZ crystal structure.

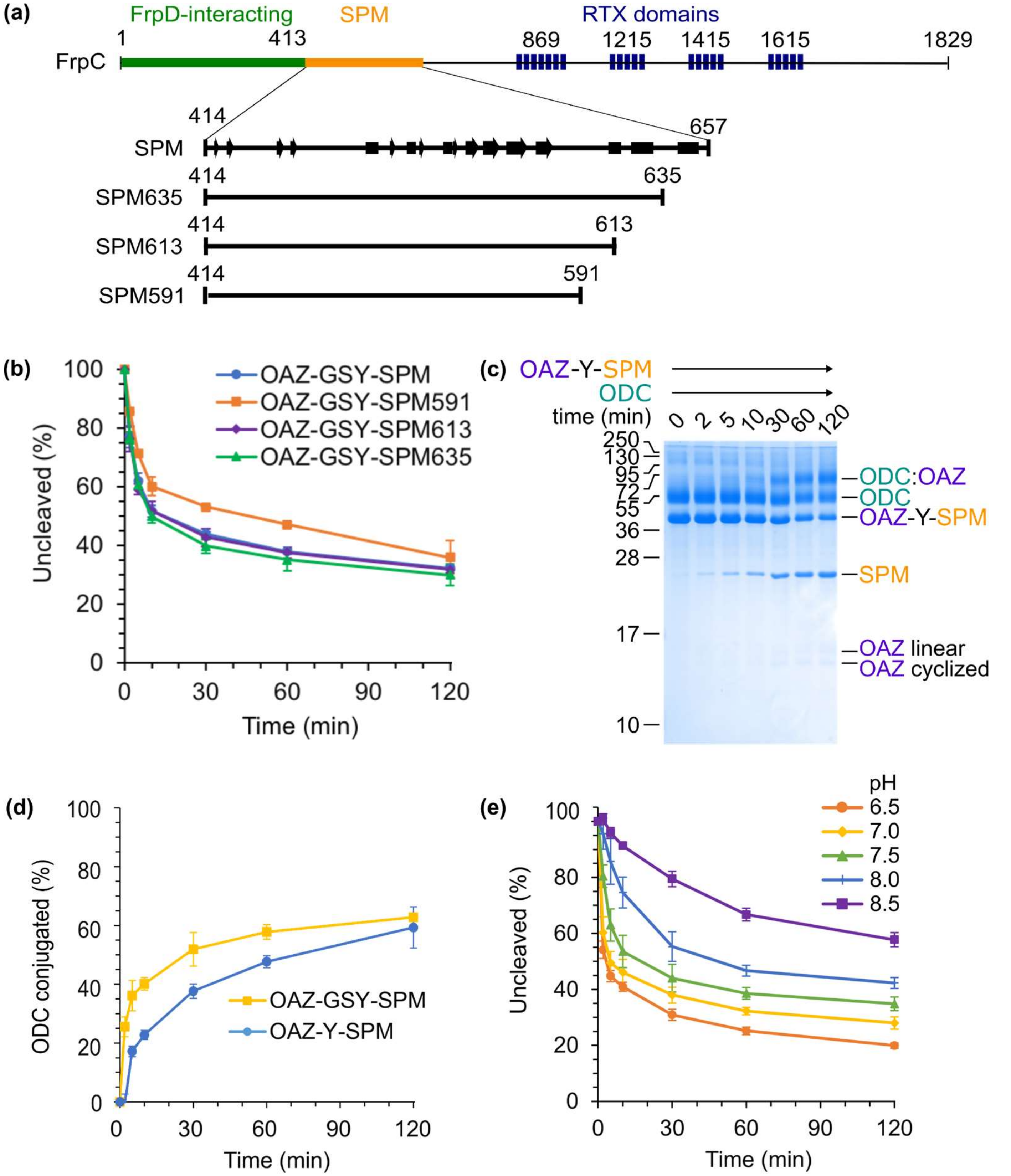

Supplementary Fig. 1. Effect of SPM truncation, linker length and pH on cleavage or conjugation rate. (a) Schematic showing truncations of SPM, alongside the regions of FrpC. Secondary structure for FrpC414-657 is from PDB 6sjw or predicted using Jpred for 591-657 (α-helix as rectangle and β-sheet as arrow). (b) Effect of SPM truncations on cleavage. 10 μM OAZ-GSY-SPM or truncated variants were incubated with 10 μM ODC in HEPES buffer at 37 °C with 10 mM Ca^{2+} for the indicated time, before analysis of cleavage by SDS-PAGE with Coomassie stain. (c) Time-course for ODC/OAZ coupling. OAZ-Y-SPM was incubated with ODC for the indicated time in the presence of Ca^{2+}, before SDS-PAGE with Coomassie staining. (d) A spacer increased conjugation rate. ODC was incubated with OAZ-Y-SPM or OAZ-GSY-SPM for the indicated time in the presence of Ca^{2+}. Coupling to ODC was determined by SDS-PAGE with Coomassie stain. (e) pH-dependence of cleavage. 10 μM OAZ-GSY-SPM was incubated with Ca^{2+} at 37 °C for varying times at the indicated pH. Analysis from SDS-PAGE with Coomassie stain. All error bars are mean of triplicate ± 1 s.d.; some error bars are too small to be visible. Source data are provided as a Source Data file.

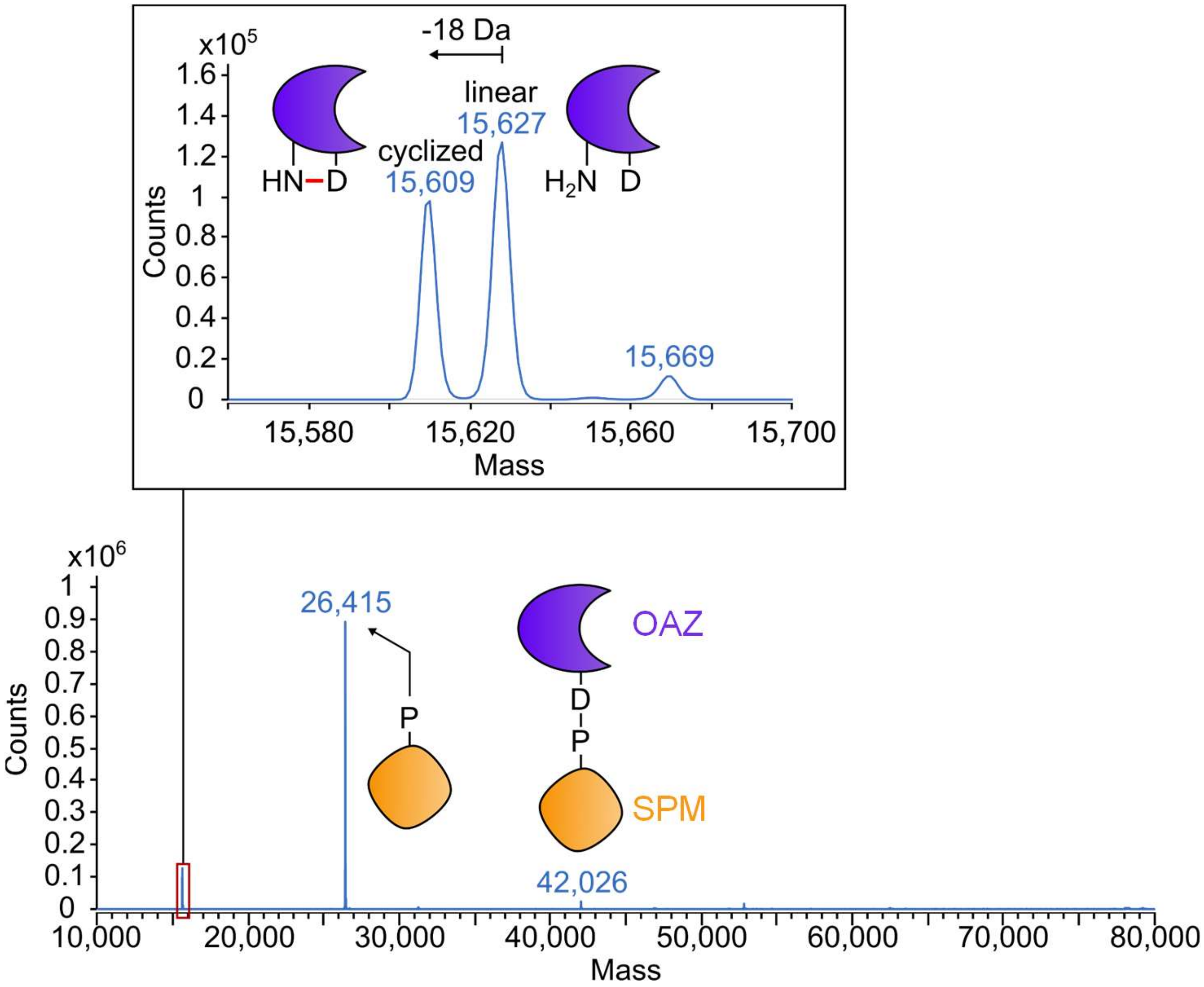

Supplementary Fig. 2. Identification of linear and cyclized OAZ species. Intact protein electrospray ionization MS following incubation of OAZ-Y-SPM in the presence of Ca^{2+}. Inset: Zoom of the red box shows linear OAZ from hydrolysis of the anhydride and a cyclized species from intramolecular reaction with the anhydride (see cartoon).

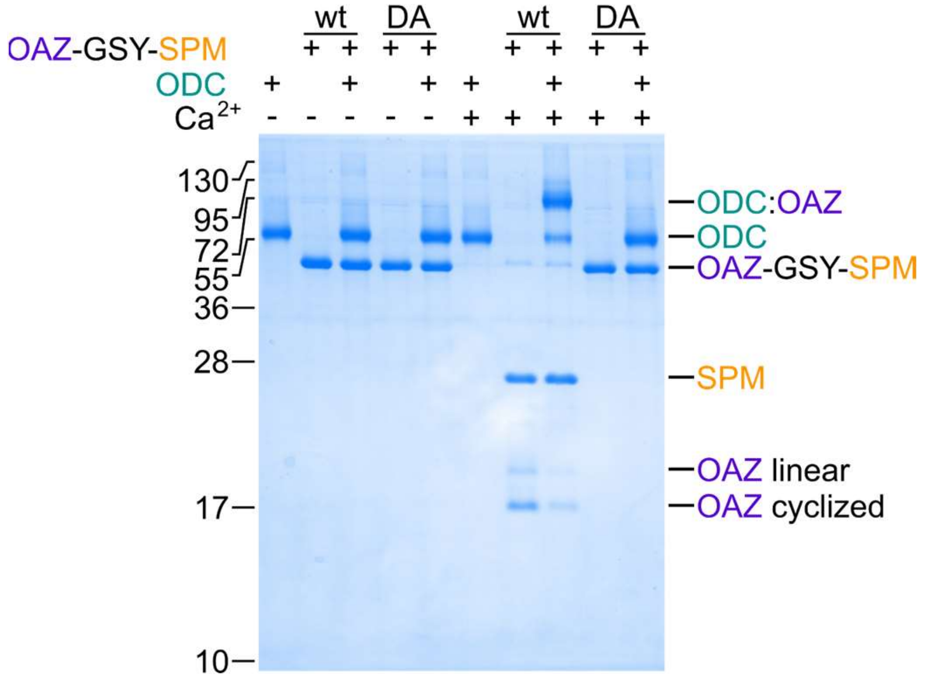

Supplementary Fig. 3. D414 in SPM was needed for cleavage and coupling.
10 µM ODC was incubated with 10 µM OAZ-GSY-SPM with or without D414A mutation at 37 °C in HBS ± 10 mM Ca^{2+}, before SDS-PAGE with Coomassie staining. Source data are provided as a Source Data file.

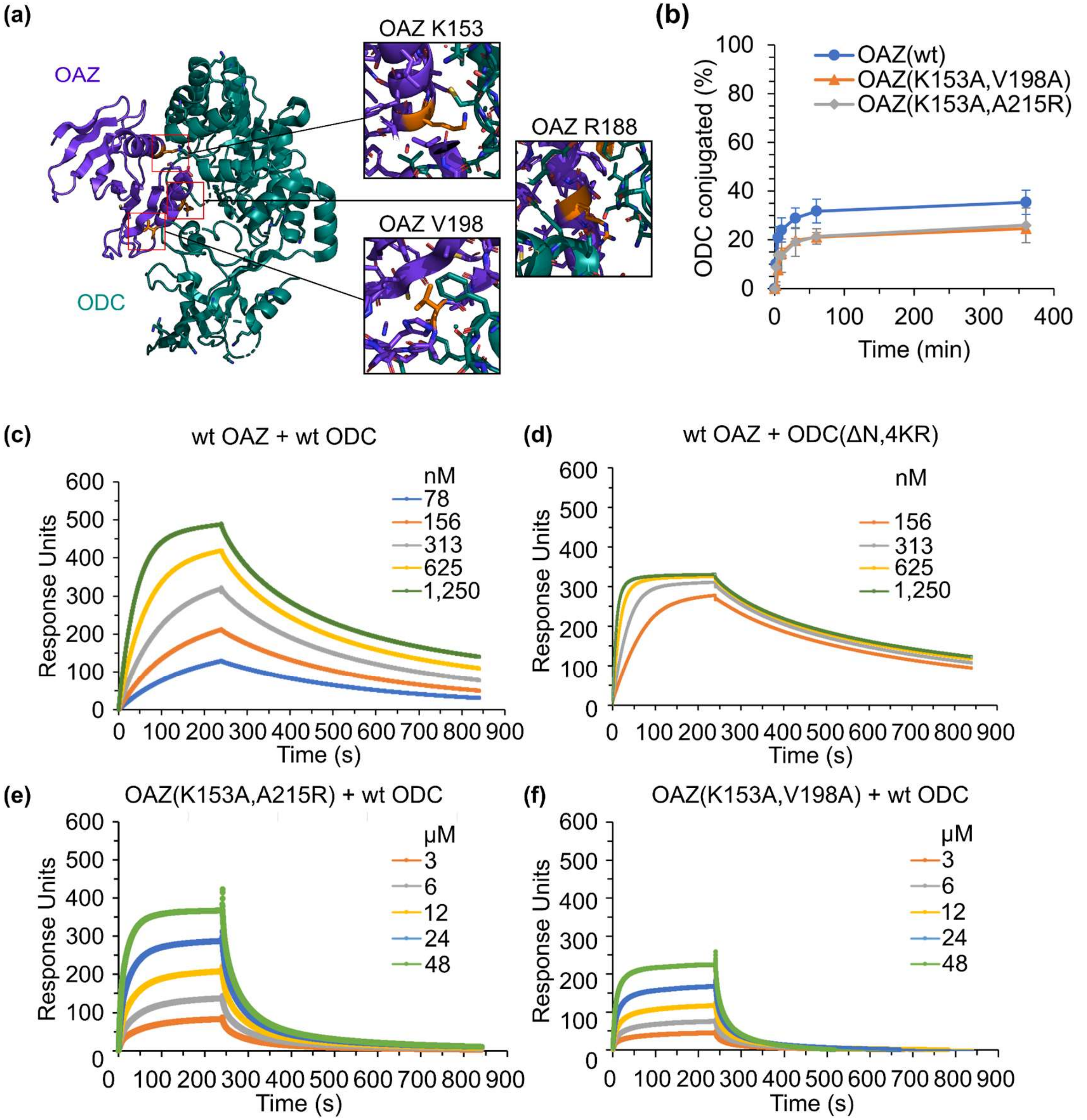

(g)

OAZ	ODC	k_{on} ($M^{-1}s^{-1}$)	k_{off} (s^{-1})	K_d (μM)
wt	wt	2.3 x 10^4	0.0028	0.12
K153A, V198A	wt	1.4 x 10^3	0.034	25
K153A, A215R	wt	1.1 x 10^3	0.017	15
K153E, R188E, V198A	wt	undetectable	undetectable	undetectable
wt	ΔN,4KR	1.1 x 10^5	0.0024	0.022

Supplementary Fig. 4. Non-covalent binding affinity between OAZ-GSY-SPM and ODC. (a) Crystal structure of OAZ:ODC complex (PDB 4zgy) with sites for K153A, R188E and V198A mutations inset and marked in orange. These mutations at the binding interface were introduced to OAZ-GSY-SPM to reduce binding affinity to ODC. (b) Conjugation rate of OAZ-GSY-SPM dual mutants to ODC (mean of triplicate ± 1 s.d.). (c-f) SPR sensorgrams for OAZ-GSY-SPM binding to ODC. Concentrations refer to ODC. Measurements were made at 25 °C without added calcium. (g) Summary of SPR data. K_d values are representative of two separate experiments. Source data are provided as a Source Data file.

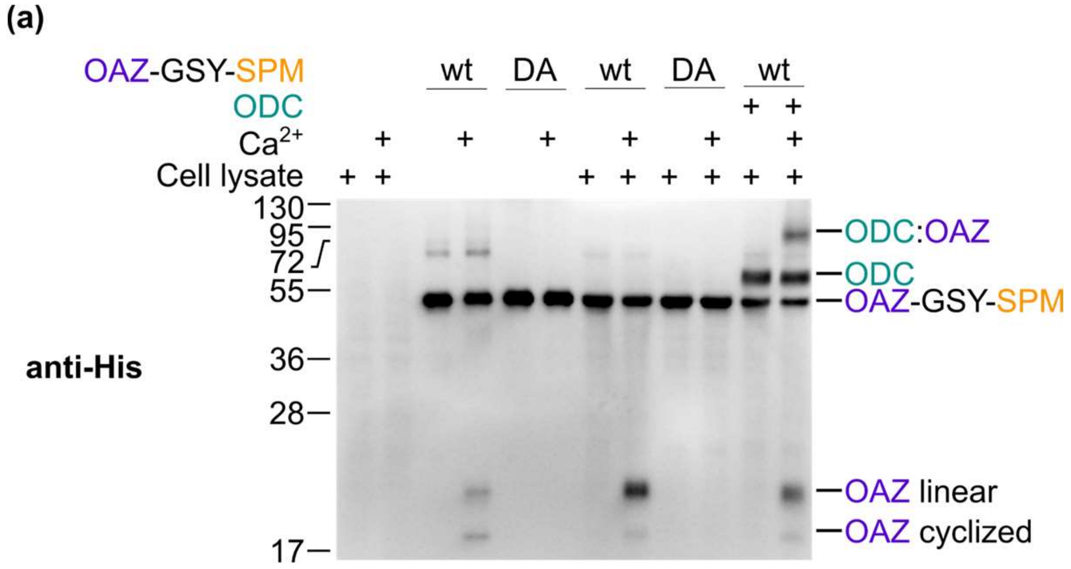

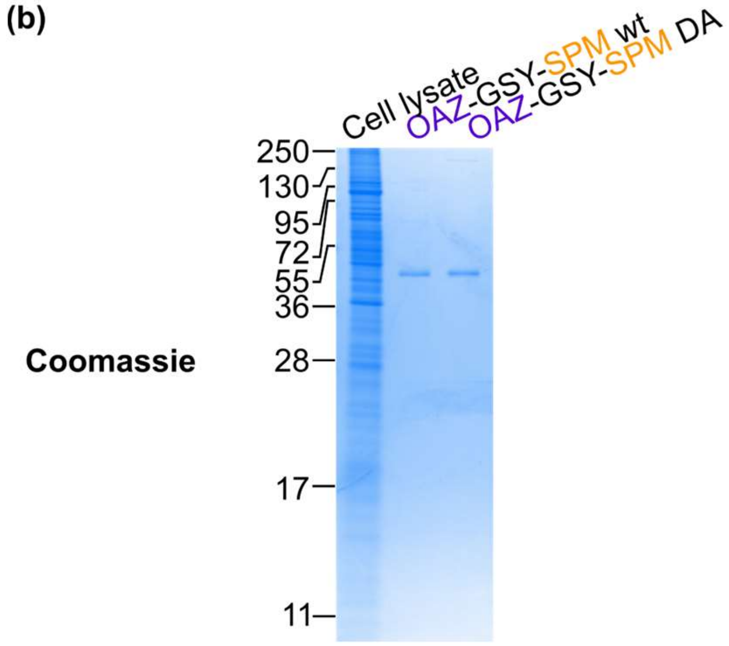

Supplementary Fig. 5. NeissLock reaction occurred in cell lysate. (a) Western blot to test the ability of OAZ-GSY-SPM to couple in cell lysate. 1 µM OAZ-GSY-SPM, with or without 1 µM ODC, was added to A431 cell lysate ± 2 mM Ca^{2+} and incubated at 25 °C for 10 min. Samples were then blotted against anti-His, detecting His_6-tags present at the N-termini of OAZ and ODC. (b) SDS-PAGE with Coomassie staining on cell lysate and wt or DA OAZ-GSY-SPM, at the same concentration as in (a). Source data are provided as a Source Data file.

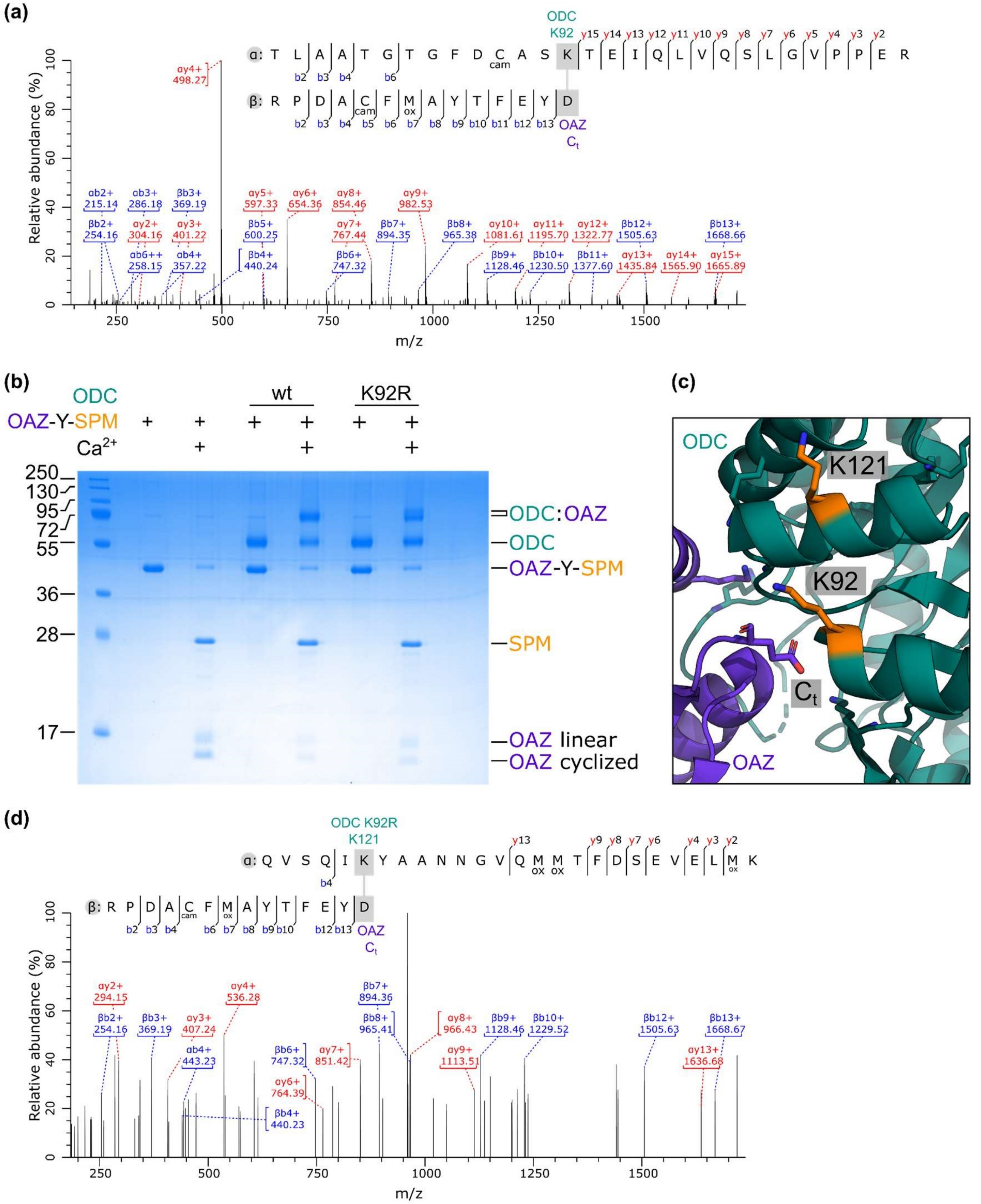

Supplementary Fig. 6. Identification of crosslinking sites for ODC reaction. (a) Tryptic LC-MS/MS of crosslink at K92 of wt ODC from reaction with OAZ-Y-SPM. (b) Corresponding SDS-PAGE with Coomassie staining for OAZ-Y-SPM coupling to wild-type or K92R ODC at 7.5 µM per protein, ± 10 mM Ca^{2+} in HBS at 37 °C for 18 h. (c) The position of K92 and K121 in the ODC/OAZ complex is shown (PDB 4zgy). (d) Tryptic LC-MS/MS of crosslink at K121 from ODC K92R reacting with OAZ-Y-SPM. cam = carbamidomethylated; ox = oxidized.

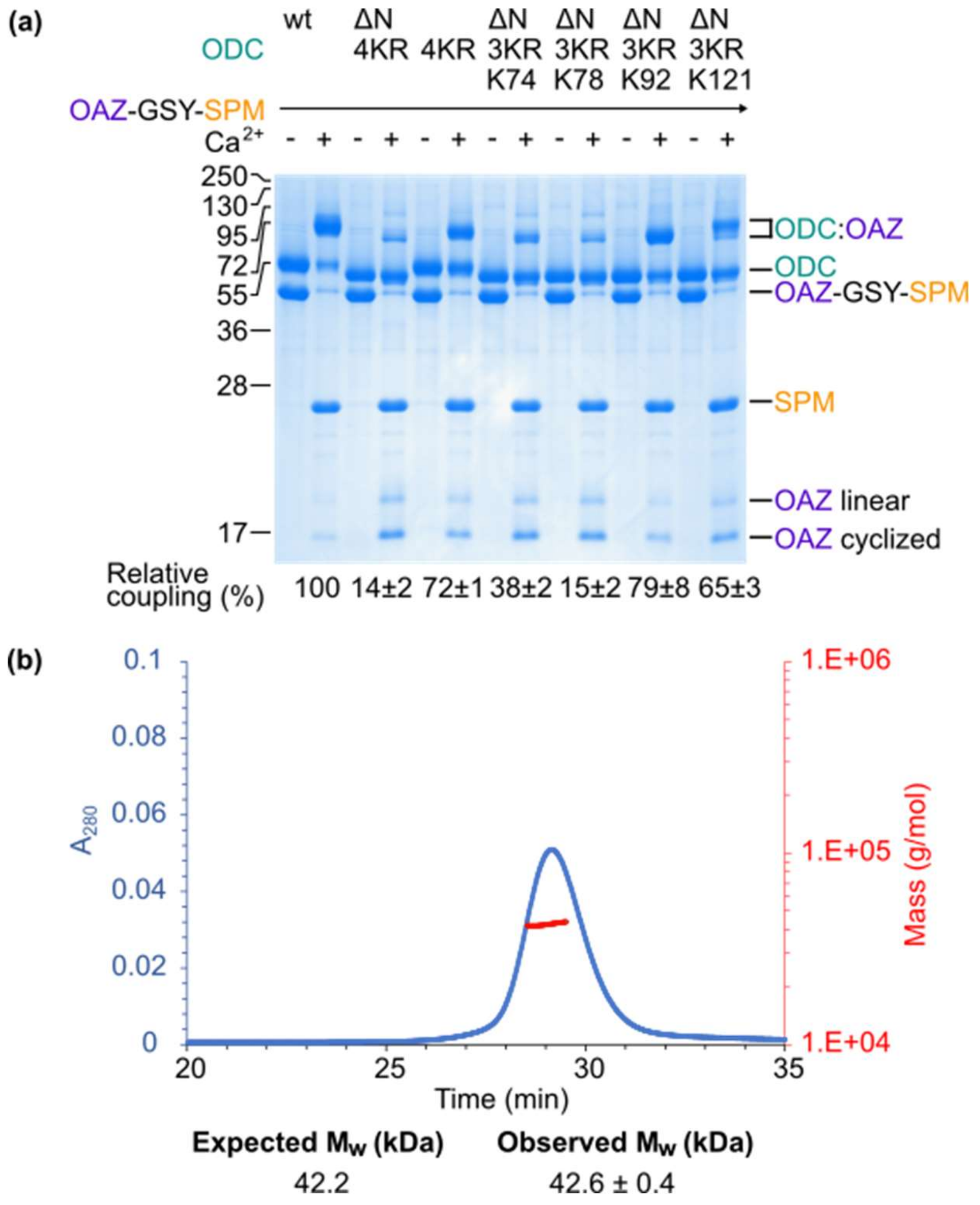

Supplementary Fig. 7. (a) Crosslinking sites in ODC. 10 μM OAZ-GSY-SPM was incubated with 10 μM of each ODC variant in HBS for 16 h at 37 °C with 10 mM Ca^{2+}, before SDS-PAGE with Coomassie staining. Conjugation is shown relative to wt ODC (mean of triplicate ± 1 s.d.). (b) OAZ-GSY-SPM was monomeric in solution when analyzed by SEC-MALS. SEC elution time is plotted against absorbance at 280 nm (blue) and MALS signal (red). Source data are provided as a Source Data file.

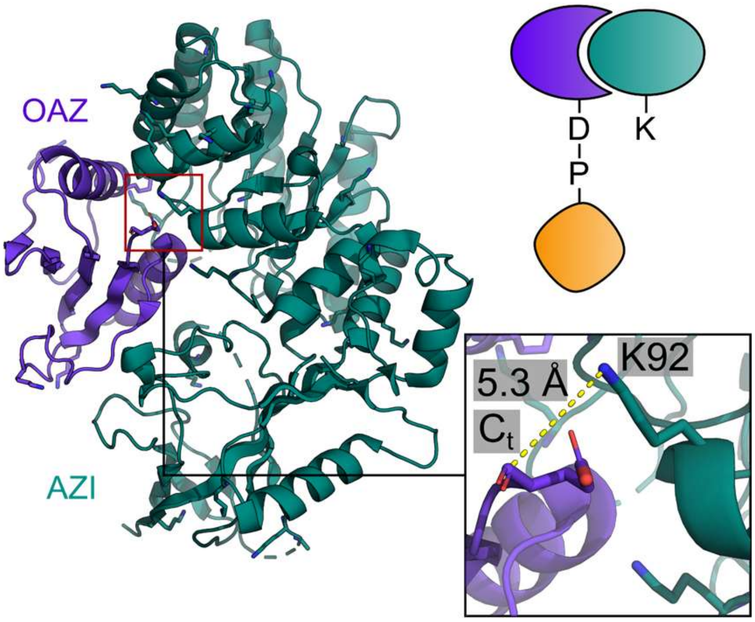

Supplementary Fig. 8. OAZ was identified as a potential NeissLock-probe to AZI. Crystal structure of AZI/OAZ complex (PDB 4zgz). The C-terminus of OAZ is in close proximity (5.3 Å) to AZI K92 ε-amine.

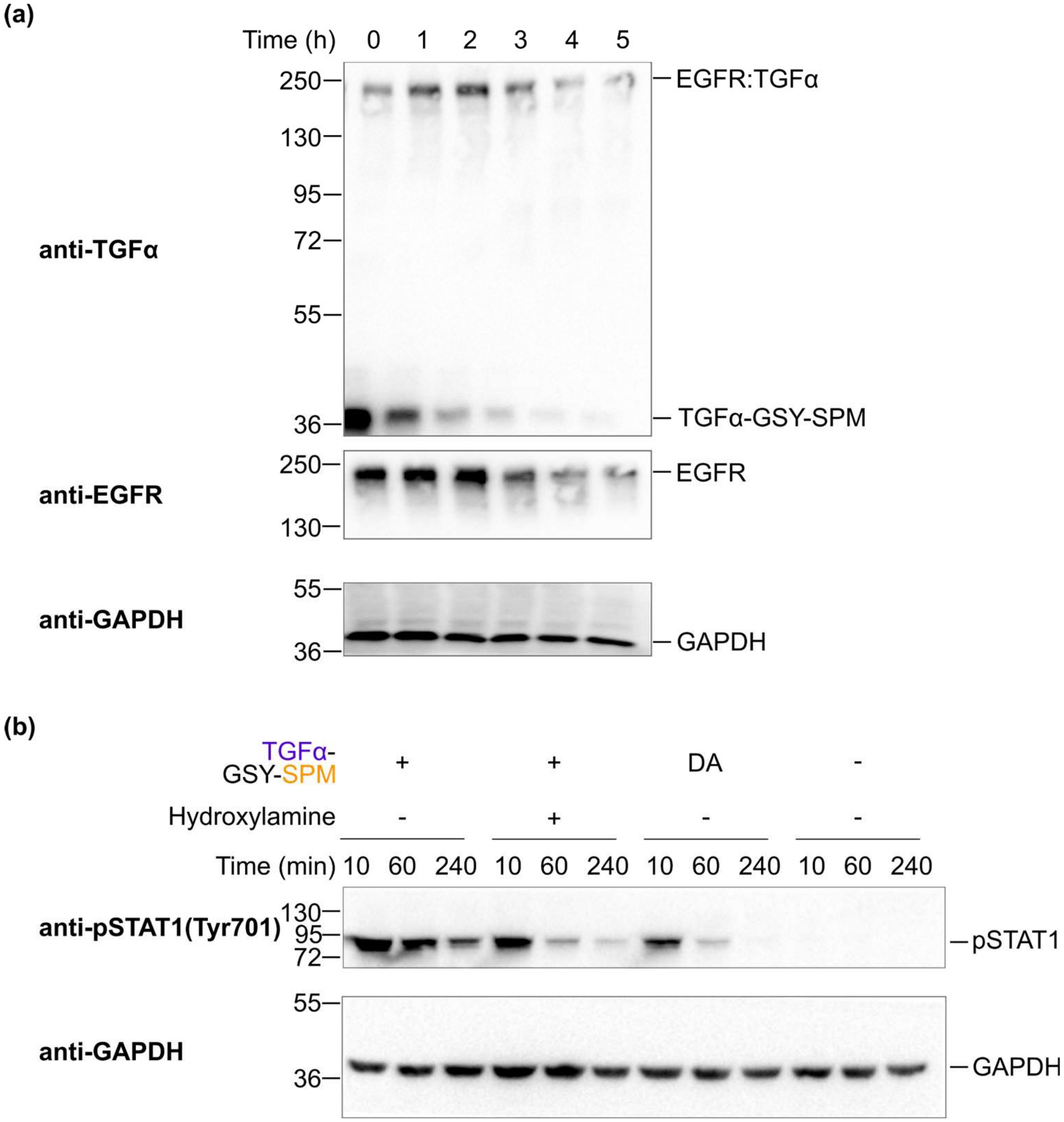

Supplementary Fig. 9. Effect of TGFα conjugation to EGFR. (a) Time-course of the EGFR:TGFα conjugate. A431 cells were mixed with 1 μM TGFα-GSY-SPM with 2 mM Ca^{2+} and then incubated with serum-free medium for the indicated time at 37 °C. Cells were blotted against TGFα or EGFR, with GAPDH as the sample processing control. (b) Covalent conjugation led to prolonged STAT1 phosphorylation. A431 cells were incubated with 1 μM TGFα-GSY-SPM or TGFα-GSY-[DA]SPM with 2 mM Ca^{2+}. Hydroxylamine was included as a control to inactivate the anhydride. Cells were then incubated with serum-free medium for the indicated time at 37 °C. Cell lysates were blotted against phosphorylated STAT1, or GAPDH as a loading control. Source data are provided as a Source Data file.
