# Supplementary material for: NeissLock provides an inducible protein anhydride for covalent targeting of endogenous proteins
Source: Nat Commun. 2021 Jan 29;12:717. doi: 10.1038/s41467-021-20963-5 (PMC7846742; doi:10.1038/s41467-021-20963-5)
Supplement: Supplementary file 2 — Description of Additional Supplementary Files [file 41467_2021_20963_MOESM2_ESM.docx]

File Name: Supplementary Data 1
Description: Resource of NeissDist database hits. All primary distances from the C-terminus to lysine residues were assessed within a given structure in the PDB. The table shows structures in which the shortest primary distance from Ct atom C to lysine atom Nε is intermolecular, <10 Å and ≥1.6 Å (to exclude covalent linkage). The table describes the name of the structure, PDB code, relevant residues, and the identified distance.

File Name: Supplementary Data 2
Description: Oligonucleotides used in this study.
